# DNA barcode assessment and population structure of aphidophagous hoverfly *Sphaerophoria scripta*: Implications for conservation biological control

**DOI:** 10.1002/ece3.6631

**Published:** 2020-08-07

**Authors:** Nemanja Gojković, Ljubinka Francuski, Jasmina Ludoški, Vesna Milankov

**Affiliations:** ^1^ Faculty of Sciences Department of Biology and Ecology University of Novi Sad Novi Sad Serbia; ^2^ Groningen Institute for Evolutionary Life Sciences University of Groningen Groningen The Netherlands

**Keywords:** integrated pest management, landscape genetics, wing geometric morphometrics

## Abstract

With the advent of integrated pest management, the conservation of indigenous populations of natural enemies of pest species has become a relevant practice, necessitating the accurate identification of beneficial species and the inspection of evolutionary mechanisms affecting the long‐time persistence of their populations. The long hoverfly, *Sphaerophoria scripta*, represents one of the most potent aphidophagous control agents due to a worldwide distribution and a favorable constellation of biological traits. Therefore, we assessed five European *S. scripta* populations by combining molecular (cytochrome *c* oxidase subunit I‐ *COI*, internal transcribed spacer 2‐ *ITS2*, and allozyme loci) and morphological (wing size and shape) characters. *COI* sequences retrieved in this study were conjointly analyzed with BOLD/GenBank sequences of the other *Sphaerophoria* species to evaluate whether *COI* possessed a sufficient diagnostic value as a DNA barcode marker to consistently delimit allospecific individuals. Additionally, the aforementioned characters were used to inspect the population structure of *S. scripta* in Europe using methods based on individual‐ and population‐based genetic differences, as well as geometric morphometrics of wing traits. The results indicate numerous shared *COI* haplotypes among different *Sphaerophoria* species, thus disqualifying this marker from being an adequate barcoding region in this genus. Conversely, the analyses of population structuring revealed high population connectivity across Europe, therefore indicating strong tolerance of *S. scripta* to environmental heterogeneity. The results imply a multilocus approach as the next step in molecular identification of different *Sphaerophoria* species, while confirming the status of *S. scripta* as a powerful biocontrol agent of economically relevant aphid pests.

## Graphical abstract

The study deals with the issue of population connectivity of an important aphidophagous hoverfly, *Sphaerophoria scripta*, based on the integrative approach—through the use of molecular and phenotypic markers, while also tackling the issue of distinguishing *S. scripta* from the congeneric species using DNA barcoding. The implications of the results are discussed in the light of pest management programs and the preservation of the ecosystem services of pest suppression and plant pollination, contributing to the growing body of knowledge concerning the protection of eco‐evolutionary interactions in times of global climate changes.

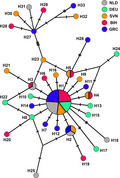

## 1 INTRODUCTION

Modern agriculture heavily relies on systematic chemical (insecticide) and mechanical (intense soil tillage) treatments to yield high productivity, consequently affecting water quality and availability, the emission of greenhouse gases, and furthermore disrupting natural ecological and evolutionary processes by causing detrimental changes in the populations of natural pest enemies (Chabert & Sarthou, 2017). Therefore, the negative consequences of contemporary agricultural practices to environmental and human health call for science‐based practices aimed at biological control of insect pests by natural enemies in agro‐ and forest ecosystems (Le Hesran, Ras, Wajnberg, & Beukeboom, 2019). The field of crop pest control saw the rise of integrated pest management (IPM), an ecosystem approach to crop production and protection that utilizes various management practices to efficiently grow high‐quality crops while minimizing the use of chemical control agents and promoting their sustainable use (Gomez‐Polo et al., 2014). One of the IPM strategies involves conservation biological control (CBC), conservation of natural enemies through manipulation of the environment. Unlike classical biological control which introduces exotic control species as agents of pest management, CBC instead involves a collection of practices which protect and promote the presence of naturally present enemies of pest species in a targeted ecosystem (Eilenberg, Hajek, & Lomer, 2001). The main advantage of CBC programs is that the beneficial species providing pest suppression service are already adapted to the habitat and to the target pest, which increases the effectiveness of the insect pest control (Gomez‐Polo et al., 2014). On the other hand, for CBC approaches to be successful, a thorough fundamental knowledge of the biology and ecology of the pest control agent is necessary. Concerning the link between natural enemy diversity and pest control (Jonsson, Kaartinen, & Straub, 2017), effectiveness of conservation biological control depends on the abundance of pest predators as well as the proper taxonomic identity of species in a predator assemblage (Moreno, Lewins, & Barbosa, 2010; Snyder, 2019). Hence, deeper understanding of the link between natural enemy biodiversity and biocontrol enhances the efficacy of conservation biological control in the management of sustainable agricultural systems (Straub, Finke, & Snyder, 2008). Furthermore, in the recent years, molecular markers have also been extensively used to elucidate various aspects of biology and ecology of beneficial species involved in biological control (MacDonald & Loxdale, 2004). One such issue includes the phenomena of population structure, tightly woven to the evolutionary mechanism of gene flow and therefore informative about the species dispersal and migratory activities (Raymond, Plantegenest, & Vialatte, 2013). Indeed, to provide sustainable biological control in the local and regional habitat, understanding the landscape effect on population connectivity of biological enemies has essential input (Happe et al., 2019). In addition, in the context of pest suppression ecosystem service, population structure is relevant for preserving the genetic diversity of the biocontrol agents (Le Hesran et al., 2019), as well as for testing their tolerance to environmental variability (Liu, Xiaoqiang, et al., 2019).

**FIGURE 1:**
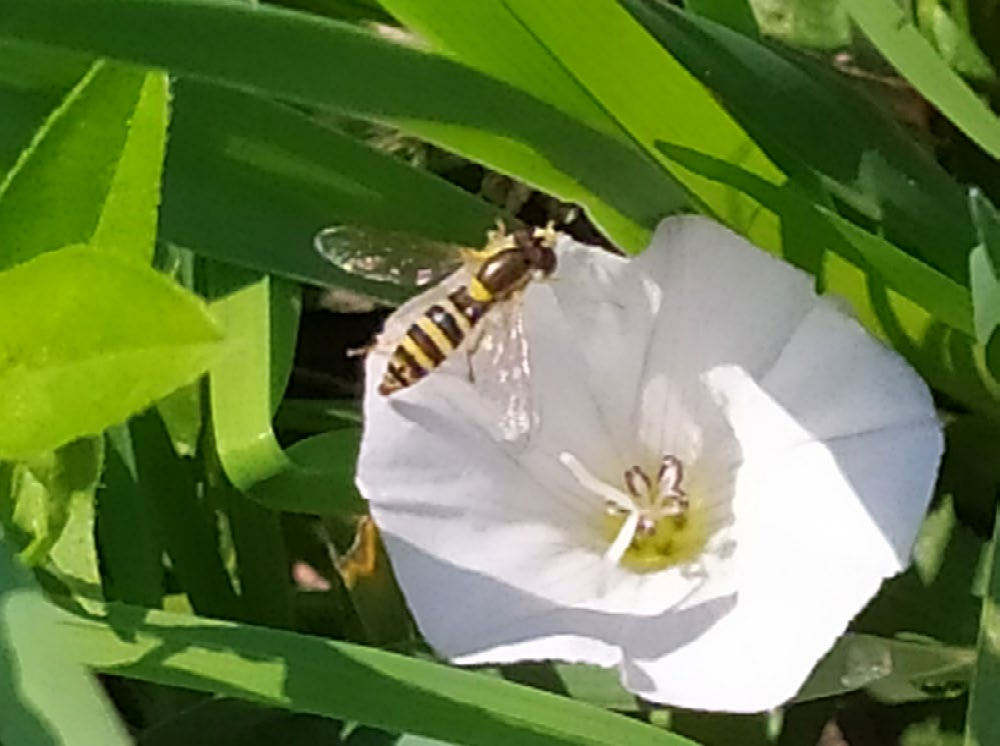
*Sphaerophoria scripta*, the long hoverfly (Diptera: Syrphidae)

Hoverflies (Diptera, Syrphidae) have recently been brought into research focus due to their key role in maintaining ecosystem services (pest control, pollination) in a diverse set of environments (Gomez‐Polo et al., 2014; Jauker & Wolters, 2008; Klecka, Hadrava, Biella, & Akter, 2018). Among the phytophagous, zoophagous, and saprophagous immatures, syrphids can be polyphagous predators of diverse arthropods, including aphids (=aphidophagous) and other economically important pest species (Gomez‐Polo et al., 2014; Rotheray & Gilbert, 1999; Speight, 2017), thereby providing the service of biological pest control. In addition, among nonbee pollinators, syrphids represent the family of most frequent visitors of the 105 major animal‐pollinated crops, also displaying a wide range of interactions by visiting over half of them (Rader, Cuningham, Howlett, & Inouye, 2020). One of the important aphidophagous syrphid groups includes the members of the Holarctic genus *Sphaerophoria* Le Peletier et Serville, 1828, which comprise 33 Palearctic species (Barkalov, 2011), 27 of which are found in Europe (Speight, 2017). Regarding the ecosystem service benefits that the *Sphaerophoria* hoverflies provide, they are an especially interesting group since predation at the larval stage and pollination at the adult stage allow a potential synergy of ecosystem services (Rader et al., 2020). Within the genus, long hoverfly, *S. scripta* (Linnaeus, 1758; Speight, 2017; Figure 1), has received particular attention as a potent biocontrol agent since it is globally distributed, characterized by intense gene flow (Raymond et al., 2013), and an abundant aphid predator constituting hoverfly larva assemblages in lettuce crops and pepper greenhouses (e.g., Pineda & Marcos‐García, 2008), as well as in other herbaceous plants (Speight, 2017). Furthermore, by boasting high genetic diversity, great colonization ability, and probably high phenotypic plasticity, long hoverfly, along with *Episyrphus balteatus* (De Geer, 1776), is considered to possess an outstanding adaptive potential with no parallel in any known arthropod species providing the same ecosystem services in agroecosystems (Raymond et al., 2013). Given that the effectiveness of CBC programs depends on the biological characteristics of the species used to suppress pest taxa (Jonsson, Wratten, Landis, & Gurr, 2008), *S. scripta* represents a potentially powerful candidate agent due to its aforementioned traits.

Over the years, several aspects of *S. scripta* traits were researched, including the following: the habitat, anatomy, and morphology of the larvae (Lal Bhatia, 1939), the abundance dynamics (Bokina, 2012; Khalil, Awadallah, & Mahmoud, 1979), the spatio‐temporal dynamics (Díaz, Legarrea, Marcos‐García, & Fereresa, 2009; Raymond et al., 2013), its parasitoids (Krawczyk, Hurej, & Jackowski, 2011), as well as the egg‐laying activities of females (Awadallah, Mahmoud, & Khalil, 1980). Additionally, the population structure of *S. scripta* was previously investigated in Europe using microsatellite loci, with the results showing no apparent stratification over a large geographic scale (Raymond et al., 2013). However, the sole use of microsatellite markers can fail to detect the existent population structure, which can be retrieved when a different marker class is used (Avise, 2000; Bernardo et al., 2019; Keeney, Heupel, Hueter, & Heist, 2005). Finally, the taxa of the hoverfly family are known to exhibit cryptic species diversity, which has been successfully revealed using DNA barcoding in several instances (Milankov, Ståhls, Stamenković, & Vujić, 2008; Ståhls et al., 2009; Šašić et al., 2016), but which has not been utilized in *S. scripta* characterization.

Since essential components of CBC involve fast and accurate identification of natural enemies, as well as the inspection of evolutionary mechanisms acting upon their populations and thereby affecting the quality of their biocontrol service, we addressed two issues of CBC in this paper: 1) the utility of DNA barcoding in the accurate identification of the taxa within the aphidophagous hoverfly genus, *Sphaerophoria*; and 2) the issue of population structure and population connectivity of polyphagous predator, *S. scripta*. We implemented several approaches using mitochondrial and nuclear genetic loci belonging to sequence and phenotypic molecular marker classes, as well as phenotypic morphological characters of adult wings.

Firstly, we used a DNA barcode approach (Hebert, Cywinska, Ball, & deWaard, 2003; Hebert, Ratnasingham, & deWaard, 2003) based on a combination of two markers frequently utilized together in insect cryptic diversity identification: a mitochondrial, cytochrome *c* oxidase subunit I gene (*COI* mtDNA) and a nuclear, internal transcribed spacer 2 locus of the ribosomal DNA cluster (*ITS2* rDNA). The DNA barcode approach relies on the existence of a barcode gap—the scenario when the greatest intraspecies haplotype divergence is lower than the least interspecies haplotype divergence (Hebert, Cywinska, et al., 2003; Hebert, Ratnasingham, et al., 2003; Puillandre, Lambert, Brouillet, & Achaz, 2011). An implicit premise to this approach, however, is that the DNA barcode method can indeed successfully distinguish between different *Sphaerophoria* species, which was also assessed by analyzing sequences belonging to 20 different *Sphaerophoria* species.

Secondly, we used clustering methods operating upon individual genetic differences to determine the most likely number of populations (=panmictic units) within the sample. Apart from *COI* mtDNA and *ITS2* rDNA, allozyme loci were also included in these analyses, with algorithms deciphering the number of populations working in two setting modes—with and without incorporating the geographic origin of the sampled *S. scripta* individuals. Additionally, different geographical samples of *S. scripta* can be a priori defined as distinct populations and the differences among them can be tested using population‐based (=group‐based) methods. This goal was reached using all the aforementioned *COI* mtDNA, *ITS2* rDNA, and allozyme loci, but also using the methodology of geometric morphometrics of adult traits (wing size and shape) as a complement to molecular markers. We opted to ascertain whether dispersal and migration of *S. scripta* individuals keep geographically remote sites genetically cohesive or whether there was a presence of population stratification, thereby affecting the co‐evolutionary dynamics of long hoverfly and the aphids it predates upon, and consequently the quality of biological pest control service it provides.

## 2 MATERIAL AND METHODS

### 2.1 Sampling and DNA barcode data set building

**FIGURE 2:**
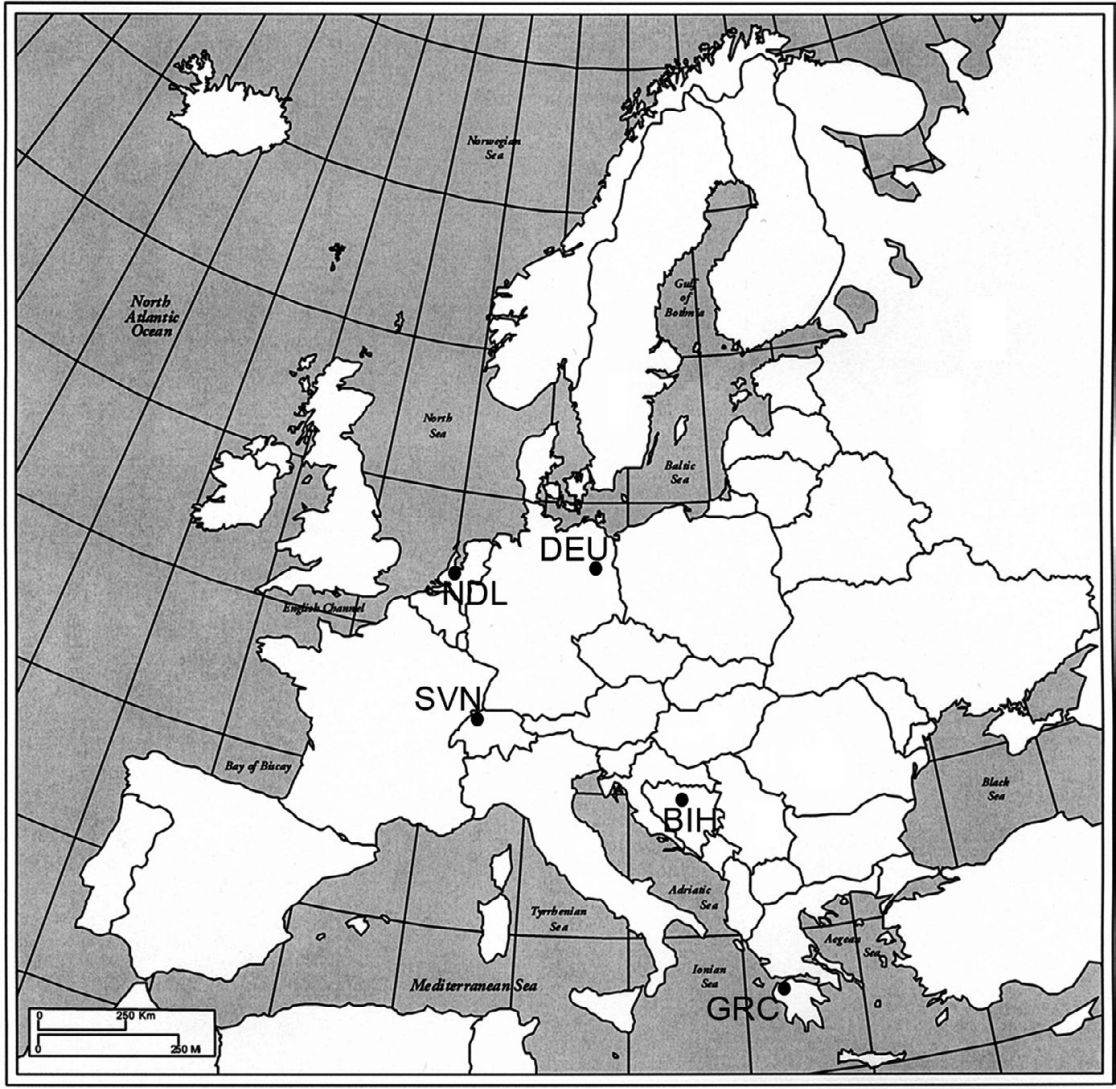
The map of the sampled localities. 1‐ the Hague, the Netherlands, NLD (52.07 N, 4.30 E), 2‐ Berlin, Germany, DEU (52.52 N, 13.40 E), 3‐ Bled, Slovenia, SVN (46.37 N, 14.11 E), 4‐ Banja Luka, Bosnia and Herzegovina, BIH (44.77 N, 17.19 E), 5‐ Araxos, Greece, GRC (38.17 N, 21.41 E). The decimal latitude and longitude geographic coordinates of the localities were retrieved from Google Earth

*Sphaerophoria scripta* specimens analyzed in this study were collected during 2010 and 2013 from five European localities: the Hague (the Netherlands; NLD), Berlin (Germany; DEU), Bled (Slovenia; SVN), Banja Luka (Bosnia and Herzegovina; BIH), and Araxos (Greece; GRC; Figure 2; leg. Milankov V, Francuski Lj, Lukač M, and Đurakić M). The adult flies were collected by hand netting while they were feeding or resting on flowers. They were subsequently stored at −20°C until the respective analyses. Prior to the analyses, the specimen identification was performed based on the morphological characters of the adults (Hippa, Nielsen, & van Steenis, 2001; det. Milankov V, Francuski Lj). In total, 154 individuals were used for geometric morphometric analyses, while a sample subset was used for the molecular analyses (Table 1). In particular, 94 *S. scripta* specimens were used in the analyses based on allozyme loci, 69 individuals for the analyses based on *COI* mtDNA, while 39 individuals were genotyped at *ITS2* rDNA locus (Table 1).

**TABLE 1:** The sampling localities and sample sizes for different *Sphaerophoria scripta* analyses performed in this study

Population (abbreviation)	Geometric morphometrics			Allozymes	*COI* mtDNA	*ITS2* rDNA
	Female	Male	Total			
the Netherlands, the Hague (NLD)	7	14	21	18	14	9
Germany, Berlin (DEU)	10	12	22	15	12	8
Slovenia, Bled (SVN)	5	17	22	21	13	7
Bosnia and Herzegovina, Banja Luka (BIH)	13	26	39	18	15	7
Greece, Araxos (GRC)	12	38	50	22	15	8
Total	47	107	154	94	69	39

Abbreviations: *COI* mtDNA, mitochondrial cytochrome *c* oxidase subunit I gene; *ITS2* rDNA, ribosomal internal transcribed spacer 2 gene.

Additionally, for the analysis of the utility of the standard DNA barcode marker (5’ end of *COI* mtDNA) for the species delimitation within the genus *Sphaerophoria*, apart from *S. scripta* sequences obtained de novo in this study, sequences deposited in BOLD and GenBank databases were also included. The databases were searched for *Sphaerophoria* genus 5’ *COI* mtDNA sequences and only the sequences belonging to the taxa identified to the species level were included. The sequences were aligned with *S. scripta COI* mtDNA sequences from this paper in BioEdit 7.0.5.3 (Hall, 1999) to choose an overlapping fragment which would be used for the subsequent analysis of the barcode utility. After the exclusion of sequences whose length was too short and/or which contained ambiguous nucleotide positions, the total data set consisted of 278 *COI* mtDNA sequences (481 bp‐long): 215 originating from BOLD database, 6 sequences downloaded from GenBank database, and 57 *S. scripta COI* mtDNA sequences from this paper (Table S1). The sequences belonged to 20 *Sphaerophoria* species, with the majority of the sample belonging to *S. scripta* (77 individuals), *S. philanthus* Meigen, 1822 (73), *S. contigua* Macquart, 1847 (50), and *S. novaeangliae* Johnson, 1916 (40; Table S1). For a subset of these sequences (Table S2), the maximum possible length of the standard 5’ *COI* mtDNA barcode (658 bp) was used to check whether the use of a shorter fragment (481 bp) affected the resolution of DNA barcoding.

### 2.2 DNA extraction and sequence processing

Total genomic DNA was extracted from single legs of the specimens using NucleoSpin® Tissue DNA extraction kit (MACHEREY–NAGEL, Düren, Germany) following the manufacturer's protocol. Polymerase Chain Reaction (PCR) using an illustra PuReTaq Ready‐To‐Go PCR Beads kit (GE Healthcare Life Sciences, Buckinghamshire, UK) was used to amplify partial sequences of *COI* mtDNA and *ITS2* rDNA genes. *COI* mtDNA sequence of 1,209 bp was obtained by overlapping amplified fragments from two separate PCR reactions: one using LCO‐1490 (5’‐GGTCAACAAATCATAAAGATATTG‐3’; Folmer, Black, Hoeh, Lutz, & Vrijenhoek, 1994)/UEA8 (alias Inger; 5′‐AAAAATGTTGAGGGAAAAATGTTA‐3′; Lunt, Zhang, Szymura, & Hewitt, 1996) primer pair and the other one using C1‐J‐2183 (alias Jerry; 5’‐CAACATTTATTTTGATTTTTTGG‐3’)/TL2‐N‐3014 (alias Pat; 5’‐TCCAATGCACTAATCTGCCATATTA‐3’) primer pair (Simon et al., 1994). *ITS2* rDNA sequence of 426 bp was amplified using ITS2A (5’‐TGTGAACTGCAGGACACAT‐3’)/ITS2B (5’‐TATGCTTAAATTCAGGGGGT‐3’) primer pair (Beebe & Saul, 1995). The total PCR volume per a sample was 25 µl, with 19 µl of ultra‐pure water, 4 µl of DNA, and 1 µl of both respective forward and reverse primers. PCR conditions were identical for all three amplification reaction types: initial denaturation at 95°C (1 min); 30 cycles of DNA denaturation at 94°C (30 s), primer hybridization with the target region at 45°C (30 s), and strand elongation at 72°C (1 min); and a final elongation at 72°C (7 min). PCR products were electrophoresed on a 2% agarose gel stained with ethidium bromide and visualized under UV light to check whether the reactions were successful. The products were subsequently purified using ExoSAP‐IT™ PCR Product Cleanup Reagent (Thermo Fisher Scientific, Vilnius, Lithuania), and bidirectionally sequenced by Macrogen (the Netherlands) on ABI3730XL using the same sets of primers used for PCRs.

Chromatograms retrieved by sequencing *COI* mtDNA (1209‐bp long) and *ITS2* rDNA (426‐bp long) loci of *S. scripta* were inspected in Chromas 2.6 (Tehnelysiumm Pty Ltd) for erroneously called bases and edited, and subsequently aligned in BioEdit. The sequences were uploaded to GenBank under the following accession numbers: MN908156–MN908221 (66 *COI* mtDNA sequences) and MN909154‐MN909155 (the two retrieved *ITS2* rDNA alleles A and B; see Results below). For three samples, only the 5’ end of *COI* mtDNA was successfully amplified (Table 1; uploaded to GenBank as MN908643–MN908645). These sequences were included in the analyses of the utility of 5’ *COI* mtDNA as a DNA barcode, but not in the individual‐ and population‐based genetic analyses which only encompassed longer *COI* sequences (Table 1). Multiple sequence alignments were performed using the ClustalW algorithm, after which the alignments were manually checked and edited when necessary. *COI* mtDNA sequences were checked for STOP codons in MEGA X 10.0.5 (Kumar, Stecher, Li, Knyaz, & Tamura, 2018) to inspect the presence of nuclear mitochondrial pseudogenes (NUMTs). The number of *COI* mtDNA haplotypes, *ITS2* rDNA alleles, and variable positions was determined in DAMBE6 (Xia, 2017). Due to the lack of variability of *ITS2* rDNA sequences (see Results below), this data set was excluded from the subsequent intended analyses.

### 2.3 Allozyme analysis

Allozyme loci of nDNA have been successfully used in hoverfly studies in a variety of subjects, including taxonomy (e.g., Milankov et al., 2008), population structure (e.g., Francuski, Ludoski, Lukac, & Milankov, 2020; Francuski & Milankov, 2015; Milankov, Ludoški, Francuski, Ståhls, & Vujić, 2013), and population genetics (e.g., Francuski et al., 2014). As a useful tool for investigating population connectivity and spatial distribution of genetic variation, the polymorphism of allozyme loci was studied by vertical polyacrylamide gel electrophoresis at eight enzyme loci: aldehyde oxidase (1.2.3.1. AO; *Ao*), α‐glycerophosphate dehydrogenase (1.1.1.8 GPD; *Gpd‐2*), β‐hydroxybutyrate dehydrogenase (1.1.1.30 HBD; *Hbd*), hexokinase (2.7.1.1 HK; *Hk‐2*), isocitrate dehydrogenase (1.1.1.42 IDH; *Idh‐1*), malate dehydrogenase (1.1.1.37 MDH; *Mdh‐2*), malic enzyme (1.1.1.40 ME; *Me*), and superoxide dismutase (1.15.1.1 SOD; *Sod‐1*). A trisborate‐ethylenediaminetetraacetic acid buffer system (pH 8.9) was used to assay AO, HK, ME, and SOD, whereas a tris‐citrate buffer system (pH 7.1) was used for analysis of GPD, HBD, IDH, and MDH. The details of buffer systems and staining procedures are given in Munstermann (1979) and Pasteur, Pasteur, Bonhomme, Catalan, and Britton‐Davidian (1988). The electrophoretic runs at 90 mA (135–220 V) lasted for 3–4 hr. The alleles of the analyzed enzyme loci were marked in an alphabetical order, where the allele encoding the electrophoretically slowest allozyme was marked as *a*. Allele frequencies were calculated in Genepop 4.7.2 (Rousset, 2008).

### 2.4 *COI* mtDNA barcode analysis

To infer whether there was cryptic diversity within the *S. scripta* specimens identified using morphological characters, we implemented a DNA barcode approach. Since this method relies on the existence of a barcode gap, if certain haplotypes are shared between two or more species, the barcode gap is closed and the marker is deemed of insufficient diagnostic utility for the species identification. Therefore, median‐joining haplotype networks were firstly constructed in NETWORK 5.0.1.1 (Fluxus Technology Ltd) to inspect the issue of shared haplotypes. The first network was constructed using solely a longer fragment of *COI* mtDNA (1,209 bp‐long) retrieved de novo for *S. scripta* specimens sampled in this study, while the second network was constructed for the total data set of *Sphaerophoria* species 5’ *COI* mtDNA sequences (481 bp‐long). When the 1,209 bp‐ and 481 bp‐long *COI* mtDNA fragments were aligned against a complete mitochondrial sequence of *Episyrphus balteatus* (GenBank accession code: KU351241), they spanned the regions corresponding to positions 1638–2846 and 1612–2092, respectively. Finally, haplotype divergences, necessary to inspect the presence of the barcode gap, were expressed as uncorrected *p* distances, where *p* is the quotient of the total number of pairwise nucleotide differences and the total length of the selected barcode region, as calculated in MEGA X 10.0.5 (Kumar et al., 2018).

### 2.5 Individual‐based clustering analyses of *Sphaerophoria scripta* specimens

The number of populations within the sample was investigated using nonspatial and spatial Bayesian clustering approaches based on individual genetic differences and implemented on both allozyme and *COI* mtDNA data sets. The nonspatial approach was implemented in BAPS 6 (Corander & Tang, 2007). For the population mixture analysis, Clustering of individuals was selected for allozyme loci, while Clustering with linked loci and Codon linkage model was used for *COI* mtDNA. For both molecular marker types, BAPS was ran for values of K (the number of genetic clusters) ranging from one to five, performing ten replicates for each K. The optimal K was selected based on the number of clusters for which the log of marginal likelihood was maximal. Mixture analysis was followed by Admixture analysis using Admixture based on Mixture results option. The number of iterations was set to 100,000, the number of reference individuals per population to 200, and the number of iterations per reference individual to 20. Additionally, STRUCTURE 2.3.4 (Pritchard, Stephens, & Donnelly, 2000) was used to evaluate the population genetic structure based on allozyme data using the admixture model with correlated allele frequencies among genetic clusters. Ten independent runs with K varying from one to five (corresponding to the five geographic samples) were performed with 10,000 generations of a burn‐in period followed by 100,000 MCMC iterations. The most probable value of K was retrieved from CLUMPAK 1.1 (Kopelman, Mayzel, Jakobsson, Rosenberg, & Mayrose, 2015) using both methods of Evano et al. (2005) and Pritchard et al. (2000), while mean population membership coefficients were obtained from STRUCTURE HARVESTER 0.6 (Earl & vonHoldt, 2012) as averaged values over ten runs for the selected value of K. To explicitly account for the sampling coordinates of the analyzed specimens, we used a spatial, individual‐based analysis in Geneland 4.0.8 (Guillot, Mortier, & Estoup, 2005; Guillot, Santos, & Estoup, 2008), as implemented in R language (v3.4.3; R Development Core Team, 2017. The number of genetic clusters was allowed to vary from one to five, with ten independent runs for each value. There were 100,000 iterations per each run, with chain thinning set to 100. Maximum rate of Poisson process was set to match the number of individuals in respective data sets (94 for allozymes, 66 for *COI* mtDNA), while maximum number of nuclei in the Poisson‐Voronoi tessellation was set to three times the number of individuals (282 for allozymes, 198 for *COI* mtDNA). No uncertainty of spatial coordinates was assumed under the correlated allele frequency model, while chain burn‐in was set to 250. Finally, to test whether a correlation between genetic and geographic distance of the sampled individuals existed, we performed Mantel's test in software Alleles in Space (AIS; Miller, 2005) using 10,000 iterations.

### 2.6 Population‐based molecular analyses of genetic differentiation

The analyses of molecular variance (AMOVA; Excoffier, Smouse, & Quattro, 1992) were performed in Arlequin 3.5.2.2 (Excoffier & Lischer, 2010) for both *COI* mtDNA and allozyme loci using standard AMOVA. Genetic differentiation was assessed among (Φ_ST_ and *F*_ST_ for *COI* mtDNA and allozymes, respectively) and between (pairwise Φ_ST_ and *F*_ST_) populations corresponding to the sampled geographical localities. To determine the statistical significance of the genetic differences, 10,000 permutations were used in permutation tests in the same software.

### 2.7 Wing geometric morphometric analysis

**FIGURE 3:**
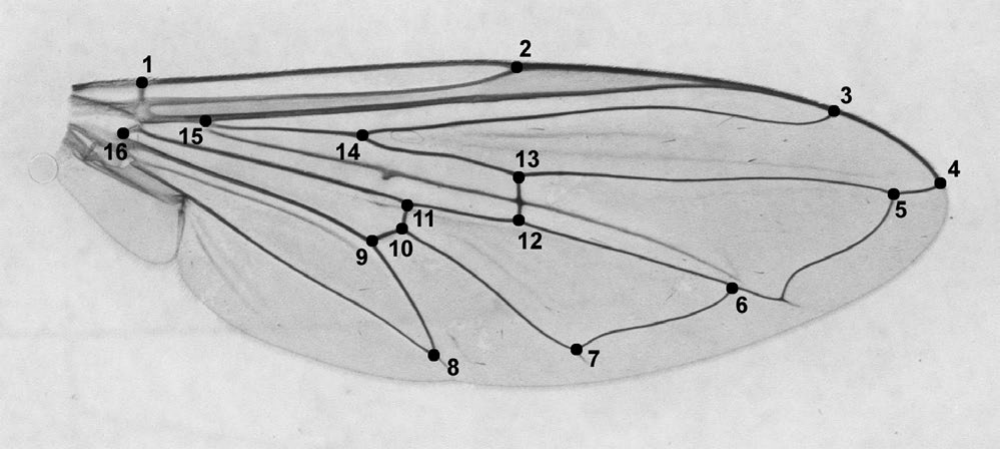
The positions of 16 landmarks used for wing size and shape geometric morphometric analysis of *Sphaerophoria scripta*

Permanent slides of both left and right wings of all 154 individuals (47 females and 107 males; Table 1) were prepared with Hoyer's medium. Right‐wing images were taken with a Leica DFC320 camera associated with a stereo microscope Leica MZ12.5 and then subjected to geometric morphometric analysis of wing size and shape. On each wing, a set of 16 landmarks were digitized using tpsDig2 ver. 2.31 (Rohlf, 2017; Figure 3). Raw landmark coordinates were superimposed using a full Procrustes fit procedure (Dryden & Mardia, 1998) and the set of shape variables (a matrix of Procrustes coordinates) and centroid size (CS; Bookstein, 1991) were extracted.

Geometric morphometric and statistical analyses were done using MorphoJ (Klingenberg, 2011) and PAST 3.26 (Hammer, Harper, & Ryan, 2001). To evaluate differences in wing size between groups (populations, sexes), we performed a permutational analysis of variance (ANOVA) on CS followed by a multiple comparison test (Tukey's pairwise post hoc test). The presence of allometry was estimated with multivariate regression of Procrustes coordinates against CS. The significance of the allometry was computed by a permutation test with 10,000 replicates. When statistically significant allometric effect was found, residuals from regression were used for the analysis of shape variation. Wing shape variation was analyzed using canonical variate analysis (CVA)/multivariate analysis of variance (MANOVA) with 10,000 permutations, while discriminant analysis (DA) was employed to calculate the percentages of correct classification, cross‐validated by a leave‐one‐out (jackknifing) procedure.

## 3 RESULTS

Among 39 individuals of *S. scripta* from the five European localities, only two alleles of *ITS2* rDNA were found; the majority of the sample was represented by an allele A (35 individuals), while an alternative allele B was registered in the remaining four individuals (Table S3). Due to the lack of variability, no further analyses on *ITS2* rDNA data set were performed.

### 3.1 Allozyme variation

Out of the eight analyzed enzyme loci, four loci were polymorphic. Up to three alleles were registered per population for the loci *Idh‐1*, *Mdh‐2*, and *Me*, while up to four alleles were registered per population for the locus *Ao* (Table S4). All the sampled individuals were homozygous for all the analyzed enzyme loci.

### 3.2 *COI* mtDNA barcode assessment

**FIGURE 4:**
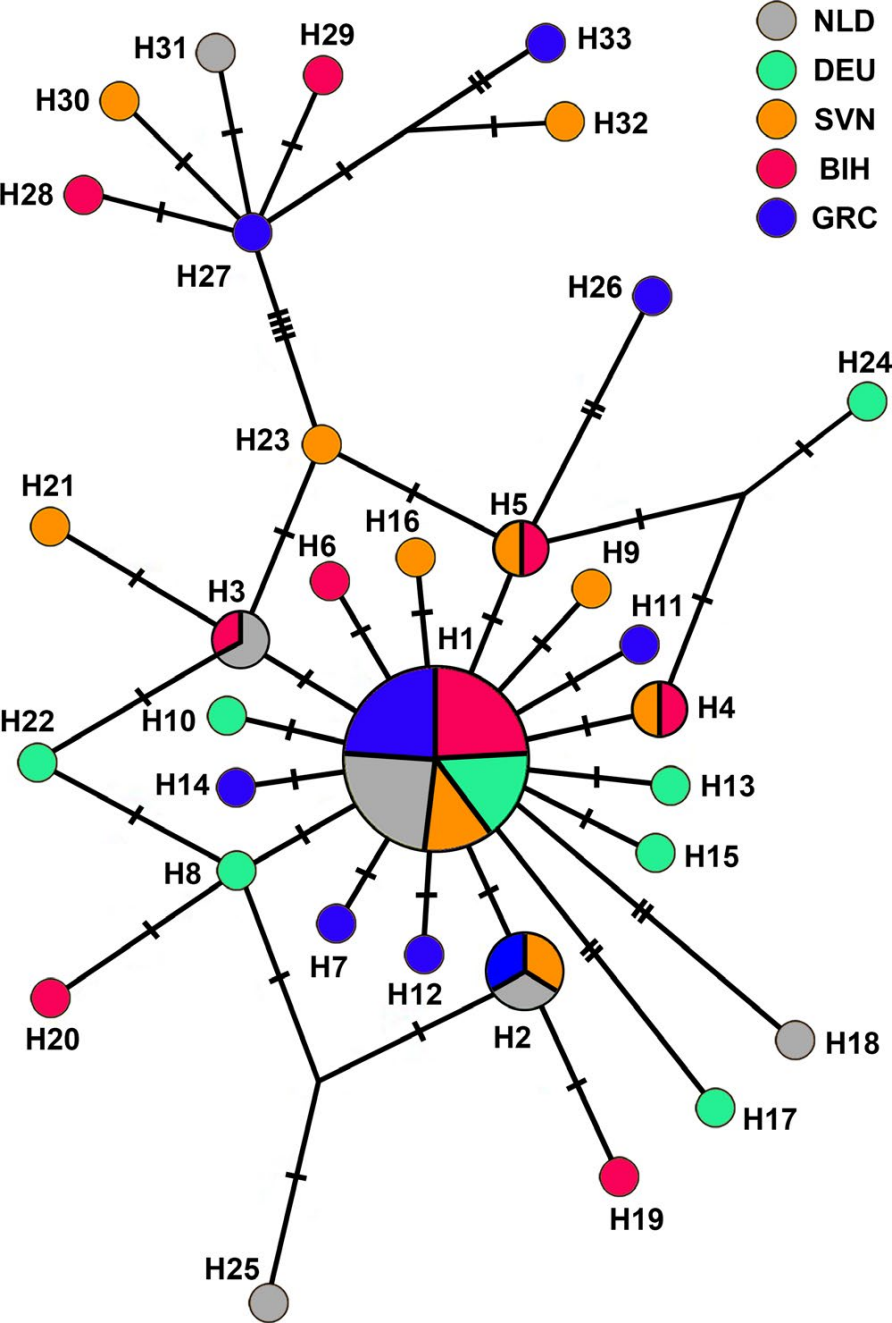
The median‐joining haplotype network of *COI* mtDNA haplotypes (1,209 bp‐long) constructed in Network. Each circle corresponds to a different haplotype, while its size is proportional to the number of individuals representing it. Dashes across lines connecting the circles indicate the number of pairwise nucleotide differences between the haplotypes, with each dash corresponding to a single nucleotide substitution. NLD—the Netherlands (the Hague), DEU—Germany (Berlin), SVN—Slovenia (Bled), BIH—Bosnia and Herzegovina (Banja Luka), GRC—Greece (Araxos)

The analysis of 66 *COI* mtDNA sequences (1,209 bp‐long) sampled from the five European localities retrieved 33 haplotypes (H1‐H33; Figure 4, Table S3), with 35 variable positions in total. The most common haplotype H1 (25/66 individuals) was present in all five geographic samples. The majority of the remaining haplotypes (H2‐H26) differed in up to three substitutions from the central haplotype H1, giving the star‐like topology to the overall haplotype network (Figure 4). BIH sample shared haplotype H3 with NLD sample, while it shared H4 and H5 with SVN sample. On the other hand, H2 was shared among NLD, SVN, and GRC, while DEU sample had no shared haplotypes with the other four countries apart from H1. Concerning the question of cryptic diversity within *S. scripta*, an additional divergent group of haplotypes originating from H27 (H28‐H33) was registered (termed the minor haplogroup as only seven haplotypes belonged to it, opposite the major haplogroup containing 26 haplotypes; Figure 4). The haplotypes within this mitochondrial haplogroup were registered in all the sampled localities, apart from Germany. The range of *p* distances was 0.08%–0.50% within the major haplogroup, 0.08%–0.33% for the minor haplogroup, and 0.33%–0.83% between the haplogroups (Table S1), therefore indicating the absence of the barcode gap since the upper range of *p* distances within the groups (0.50%) was greater than the lower range between the groups (0.33%). However, when the *COI* mtDNA sequences were aligned against a complete mitochondrial sequence of *Episyrphus balteatus*, the haplotypes of the minor haplogroup were characterized by a diagnostic combination of six substitutions: 1,783 T, 1,978 G, 2,495 G, 2,500 T, 2,734 C, and 2,761 G (Figure S1). Therefore, despite the absence of the distance‐based barcode gap between the haplogroups, the members of the minor haplogroup were consistently distinguished from the major haplogroup haplotypes by a specific combination of diagnostic nucleotide states (the six aforementioned substitutions). To further explore this issue and investigate whether the minor *S. scripta* haplogroup indeed could represent a distinct taxon, DNA barcode analysis was implemented on the total *Sphaerophoria* genus data set.

**FIGURE 5:**
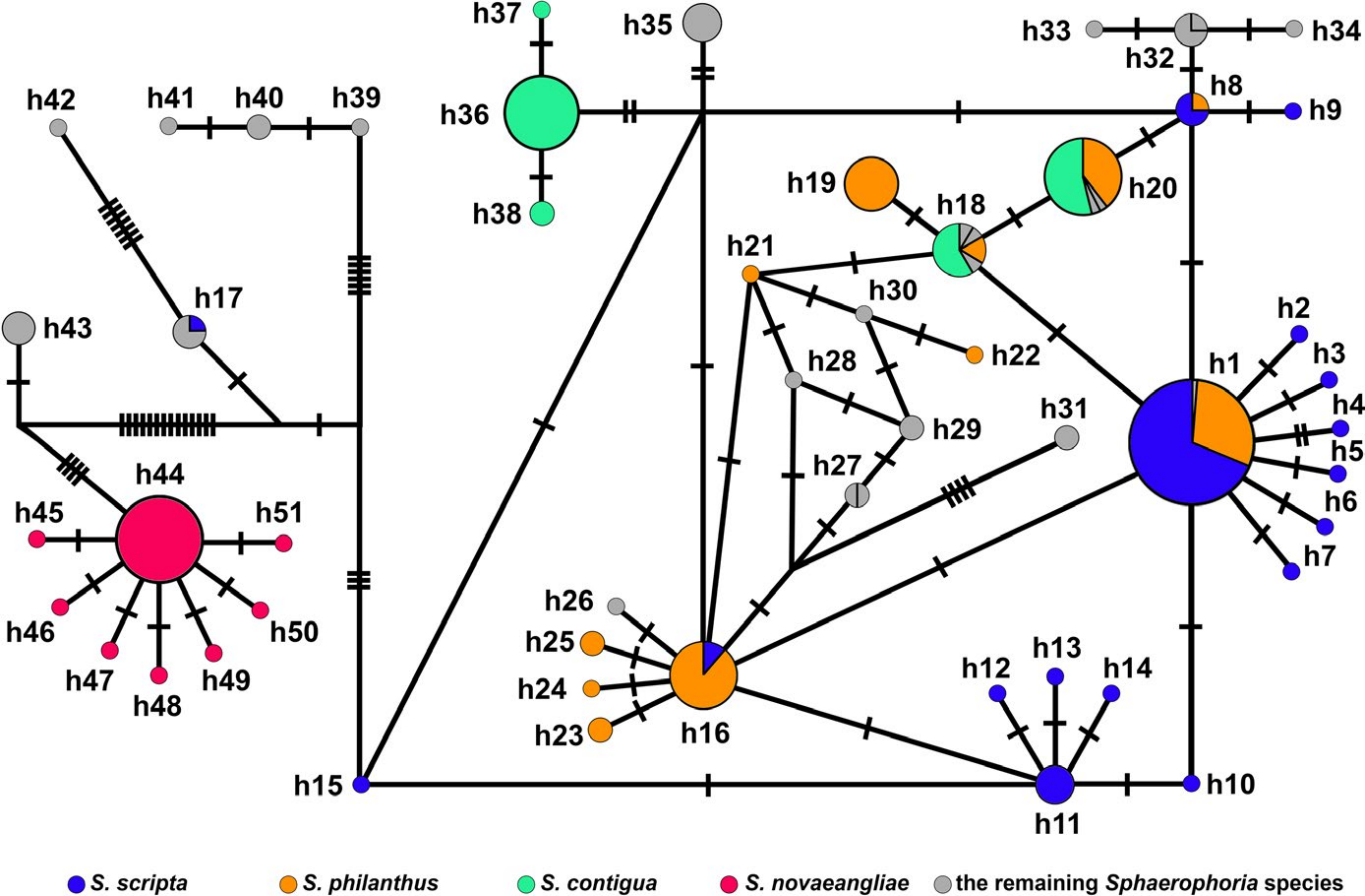
The median‐joining haplotype network of 5’ *COI* mtDNA haplotypes (481 bp‐long) constructed in Network. Out of the 20 analyzed *Sphaerophoria* species (Tables S1 and S3), the four which mostly contributed to the sample size are distinctly colored, while the remaining species share the same color. Each circle corresponds to a different haplotype, while its size is proportional to the number of individuals representing it. Dashes across lines connecting the circles indicate the number of pairwise nucleotide differences between the haplotypes, with each dash corresponding to a single nucleotide substitution

The total data set analysis of 278 5’ *COI* mtDNA sequences (481 bp‐long) of 20 *Sphaerophoria* species retrieved 51 haplotypes (h1‐h51; Figure 5, Tables S3 and S5), with 58 variable positions. Concerning the haplotypes belonging to the minor *S. scripta* haplogroup (H27‐H33), they were positioned as a group of haplotypes oriented around haplotype h11: H31‐H33 corresponded to h11, H29 to h13, H30 to h14, and H28 to h15 (H27 was excluded from this analysis due to being too short at the 5’ end). Apart from these sequences, retrieved from our study, BOLD database sequences belonging to this haplogroup (h11–h15) were additionally retrieved from Greenland (h11 and h12) and Norway (h11). Therefore, the representatives of the minor *S. scripta* haplogroup were codistributed with the members of its major haplogroup, being present in Greenland and from Norway in the north to Greece in the south in Europe, spanning over the Netherlands, Slovenia, and Bosnia and Herzegovina. To conclude whether this set of haplotypes represented a putative distinct species depended on whether 5’ *COI* mtDNA represented a diagnostic DNA barcode marker for identification of different *Sphaerophoria* species. However, from the barcode utility standpoint, the partial sequence of the standard 5’ *COI* mtDNA marker failed to successfully delimit different *Sphaerophoria* species. In particular, there were eight haplotypes which were shared between two or more allospecific individuals: h8, h16, h17, h27, and h32 were common for two respective species, the most frequent haplotype h1 was shared among three species, h20 was retrieved from individuals belonging to four different species, while h18 was present in five different *Sphaerophoria* species (Table S5). Since the existence of barcode gaps depends on the condition of the greatest intraspecies divergence being lower than the smallest interspecies divergence, when two species share certain haplotypes (the lowest interspecies divergence: *p* = 0%; see Table S2), the barcode gap is sealed and the analyzed marker is deemed unsuccessful for the species delimitation. Speaking strictly for the four species which contributed to the majority of the sample, *S. philanthus* and *S. contigua* shared haplotypes h18 and h20, while *S. philanthus* and *S. scripta* shared haplotypes h1, h8, and h16. On the other hand, *S. novaeangliae* had no haplotypes in common with the other *Sphaerophoria* species, meaning that 5’ *COI* mtDNA possesses a limited diagnostic power for congeneric species identification, being successful in this case (Figure 5, Table S2).

Prior to definitively concluding about the suitability of 5’ *COI* mtDNA for the delimitation of different *Sphaerophoria* species, we considered whether the use of the shorter fragment significantly affected the outcome of species delimitation. Namely, for the subset of sequences representing the eight shared haplotypes (149 sequences, Table S2), we performed the alignment of their full‐length sequences to inspect whether possibly new diagnostic positions would arise that were previously omitted when the shorter fragment was used. Out of the 149 sequences, 73 sequences contained the entire 658 bp‐long barcode content, 58 contained at least 600 bp of the standard barcoding fragment, while the remaining 18 missed 120 bp at most. Within this data set, five additional variable positions were registered (Table S2), separating some of the previously identical sequences. However, apart from haplotype h8, the pattern of the lowest interspecies *p* divergence being 0% persisted, with even 658 bp‐long sequences of different *Sphaerophoria* species remaining identical. Specifically, h1 and h16 were shared between *S. scripta* and *S. philanthus*, h17 between *S. scripta* and *S. rueppellii*, h20 between *S. philanthus* and *S. contigua*, h27 between *S. taeniata* and *S. interrupta*, h32 between *S. virgata* and *S. laurae*, and finally—h18 being shared among *S. philanthus*, *S. contigua*, *S. batarum*, and *S. asymmetrica* (Table S2). Summarily, the resolution of 5’ *COI* mtDNA as a DNA barcode marker in *Sphaerophoria* genus was not significantly affected by the use of its shorter (481 bp) fragment and remained unsuitable. Therefore, it was not possible to unequivocally confirm or dismiss whether the members of the minor *COI* mtDNA haplogroup of *S. scripta* represented a distinct taxon using the DNA barcode approach (but see Discussion), and neither was it possible to use this DNA barcode marker to distinguish different *Sphaerophoria* genus species.

### 3.3 Individual‐based genetic clustering analyses

For *S. scripta COI* mtDNA data set, BAPS retrieved three as the most probable number of clusters. The majority of the haplotypes (19/33) were grouped together in cluster K1, while haplotypes H27‐H33, belonging to the minor *COI* mtDNA haplogroup, were recognized as a separate genetic cluster (K3). The remaining haplotypes were grouped together in a cluster K2 which included two separate subgroups—K2a, whose haplotypes (H5, H23, H24, H26) were grouped by having a common 2,761 G substitution, and K2b, whose haplotypes (H2, H19, H25) shared a 2,788 T substitution (Figure S1). The populations sampled in the Netherlands, Germany, and Slovenia had high average population membership probabilities to cluster K1 (>80%, Table S5). The remaining two populations had relatively lower average population membership probability to K1 (around 50%), with the remaining membership probabilities distributed between K2 and K3 (43% and 7% for Bosnia and Herzegovina, respectively, and 32% and 20% for Greece, respectively; Table S6). However, since there was no biological rationale for establishing separate clusters K1 and K2 within the major *COI* mtDNA haplogroup (see Discussion below), BAPS analysis solely confirmed the existence of the two separate haplogroups within the sample, with the sequences belonging to the major haplogroup being recognized as K1 and K2, while the sequences belonging to the minor haplogroup were clustered together in K3. Importantly, these two clusters were geographically codistributed (apart from Germany where K3 was absent) and the majority of the sample had negligible contribution of K3 to its structure, meaning that the five geographically distinct samples actually represented a homogenous genetic sample, predominantly being constituted from the members of the major *COI* mtDNA haplogroup (>80% for each sample when K1 and K2 are added together). This was also supported upon the incorporation of spatial data in Geneland, where a homogenous sample was found, without detecting any structuring (K = 1). Finally, Mantel's test, investigating the correlation between genetic and geographic distances, yielded statistically nonsignificant results (*r* = −.03, *p* = .69), showing no isolation by distance pattern. All three analyses—BAPS, Geneland, and Mantel's test, were repeated upon the exclusion of the members of K3 and the identical results were retrieved, meaning that the incorporation of K3 members did not obscure the pattern of no population structuring.

**FIGURE 6:**
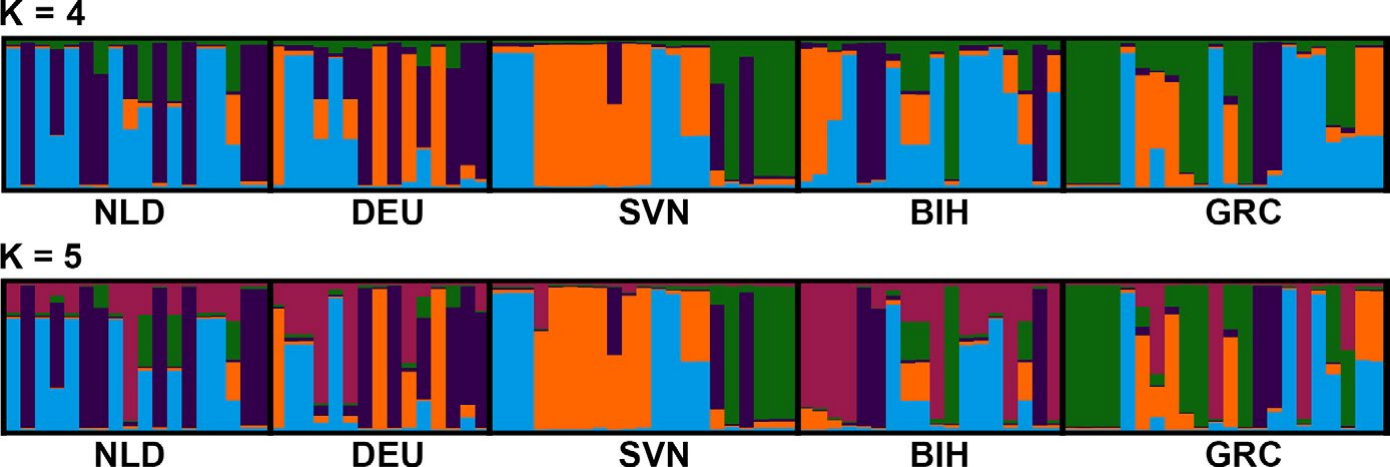
Population genetic structure analysis of the five European *Sphaerophoria scripta* populations using STRUCTURE software on allozyme loci. The most probable number of clusters varied between the two methods‐ Evano et al. (2005) and Pritchard et al. (2000), corresponding to K = 4 and K = 5, respectively. Regardless of the most probable number of homogenous genetic groups within the sample, both scenarios indicate a pattern of high genetic admixture. NLD—the Netherlands (the Hague), DEU—Germany (Berlin), SVN—Slovenia (Bled), BIH—Bosnia and Herzegovina (Banja Luka), GRC—Greece (Araxos)

Concerning the allozyme loci, Bayesian clustering method implemented in BAPS retrieved five as the most probable number of clusters. However, neither of the geographic samples was particularly differentiated as belonging to any of the genetic clusters (the highest average membership probability to any of the clusters was lower than 45%; Table S6). Furthermore, STRUCTURE retrieved four and five as the most probable number of homogenous genetic groups within the total sample using the methods of Evano et al. (2005) and Pritchard et al. (2000), respectively (Figure 6). Once again, regardless of the value of K, neither of the geographic samples had significantly high mean population membership coefficient to the retrieved genetic clusters (threshold value is usually taken at 80%, but no locality had membership coefficient higher than 46%; Table S7). Therefore, all the geographic samples were genetically admixed. The inclusion of spatial data in Geneland indicated four as the most probable number of clusters, with Slovenia, Bosnia and Herzegovina, and Greece representing discrete samples, while the northernmost samples—the Netherlands and Germany, were grouped together. Finally, Mantel's test results showed no statistically relevant correlation between genetic and geographic distances (*r* = .03, *p* = .15).

### 3.4 Population‐based molecular analyses of genetic differentiation

The analysis of molecular variance based on *COI* mtDNA found no significant Φ_ST_ value for the *S. scripta* geographic samples (*p* = .74; Table S8). Similarly, there were no statistically significant pairwise Φ_ST_ values, overall indicating no population structuring within the analyzed data set. The same results were obtained upon the repetition of the analyses without the sequences belonging to the minor *COI* mtDNA haplogroup (H27‐H33). Conversely, interpopulation differences were small yet significant when allozyme loci were analyzed, with *F*_ST_ = 0.08 (*p* = .02; Table S8). Furthermore, pairwise *F*_ST_ values were significant for Slovenia/Bosnia and Herzegovina and Slovenia/Greece population pairs (*p* < .05; Table S9).

### 3.5 Wing geometric morphometrics

Prior to wing shape variation analysis, multivariate regression of shape variables on CS was performed for a total sample, as well as for each sex separately. In an overall sample, 7.2% (*p* < .0001) of shape variation was explained by size, while it was 4.0% (*p* = .05) in females and 10.2% (*p* < .0001) in males. Thus, due to the presence of allometry, regression residuals were used for further analysis of shape variation.

**FIGURE 7:**
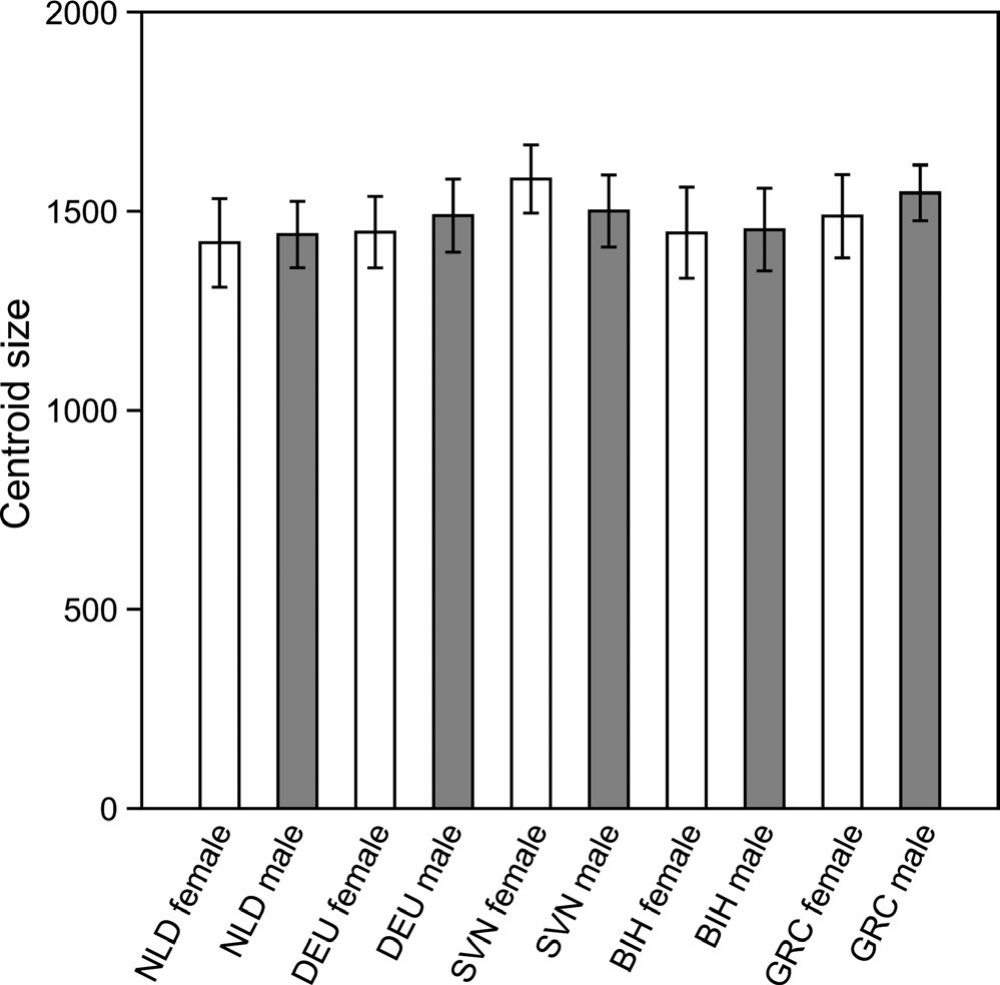
Wing size variation within and between populations of *Sphaerophoria scripta*. The whisker interval represents standard deviation of the mean. NLD—the Netherlands (the Hague), DEU—Germany (Berlin), SVN—Slovenia (Bled), BIH—Bosnia and Herzegovina (Banja Luka), GRC—Greece (Araxos)

**FIGURE 8:**
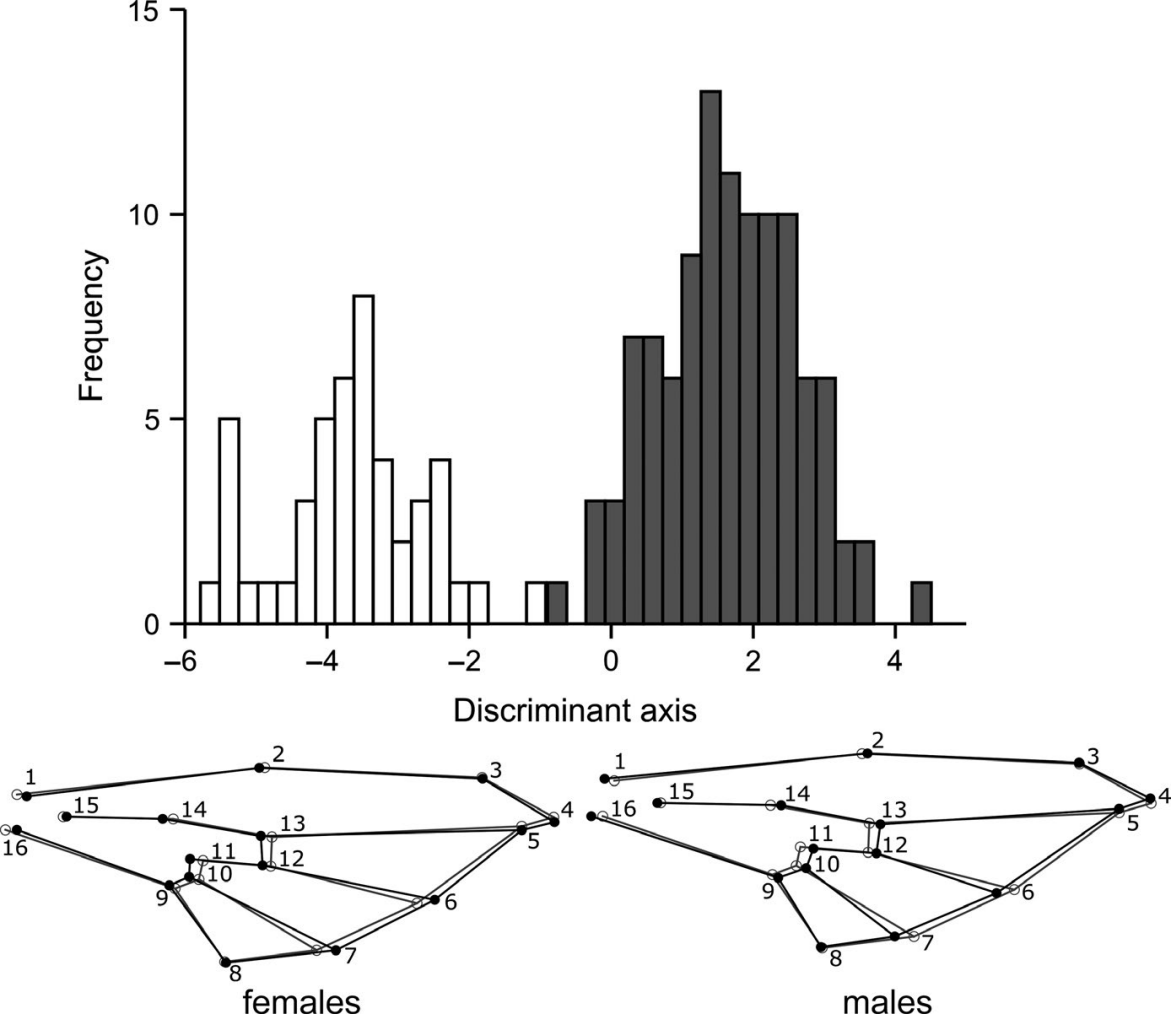
Wing shape differentiation between females (white bars) and males (gray bars) of *Sphaerophoria scripta* obtained with DA. Wing shape changes (black lines) from consensus configuration of landmarks (gray lines) along the discriminant axis are shown using the wireframe graphs (numbers refer to landmarks shown in Figure 2)

Two‐way ANOVA with dependent variables of size (CS) and independent variables of population and sex revealed a significant main effect of population (*F*_(4, 144)_=5.47; *p* < .0001) and interaction (*F*_(4, 144)_ = −6.92; *p* = .03; Table 2; Figure 7). Two‐way MANOVA on regression residuals showed significant main effect of population (*F*_(4, 144)_ = 1.51; *p* < .001) and sex (*F*_(4, 144)_ = 49.52; *p* < .0001; Table 2). Additionally, DA revealed that males and females clearly separated along the discriminant axis (Procrustes distance = 0.0312; *p* < .0001) with 100% (98.7% jackknifed) of correct classification. Displacement of landmarks 9–14 being moved toward either distal or proximal part of wing in males and females, respectively, contributed to intersexual wing shape differences (Figure 8). Due to significant differences between genders, further wing size and shape variation analyses were done for each sex separately.

**TABLE 2:** The results from two‐way ANOVA/MANOVA on wing size (centroid size, CS) and shape (residuals from multivariate regression) variables, respectively, representing variation of wing traits between and within populations of *Sphaerophoria scripta*

	SS	*df*	MS	*F*	*p*
Two‐way ANOVA on CS					
Population	237,070	4	59,267	5.47	<.0001
Sex	26,778	1	26,778	2.47	.07
Population × sex	−299,770	4	−74,941	−6.92	.03
Within‐group	1,560,400	144	10,836		
Total	1,524,500				
Two‐way PERMANOVA on size‐corrected shape variables (regression residuals)					
Population	0.003875	4	0.000969	1.51	<.001
Sex	0.031864	1	0.031864	49.52	<.0001
Population × sex	−0.027084	4	−0.006771	−10.52	.77
Residual	0.092662	144	0.000644		
Total	0.101322				

ANOVA performed on centroid size revealed significant interpopulation differences for males (*F*_(4, 102)_ = 6.11, *p* < .001) but not for females (*F*_(4, 42)_ = 2.19, *p* = .09). Tukey's post hoc test showed that males from GRC were significantly larger than males from NLD (*p* < .001) and BIH (*p* < .001; Figure 7).

**FIGURE 9:**
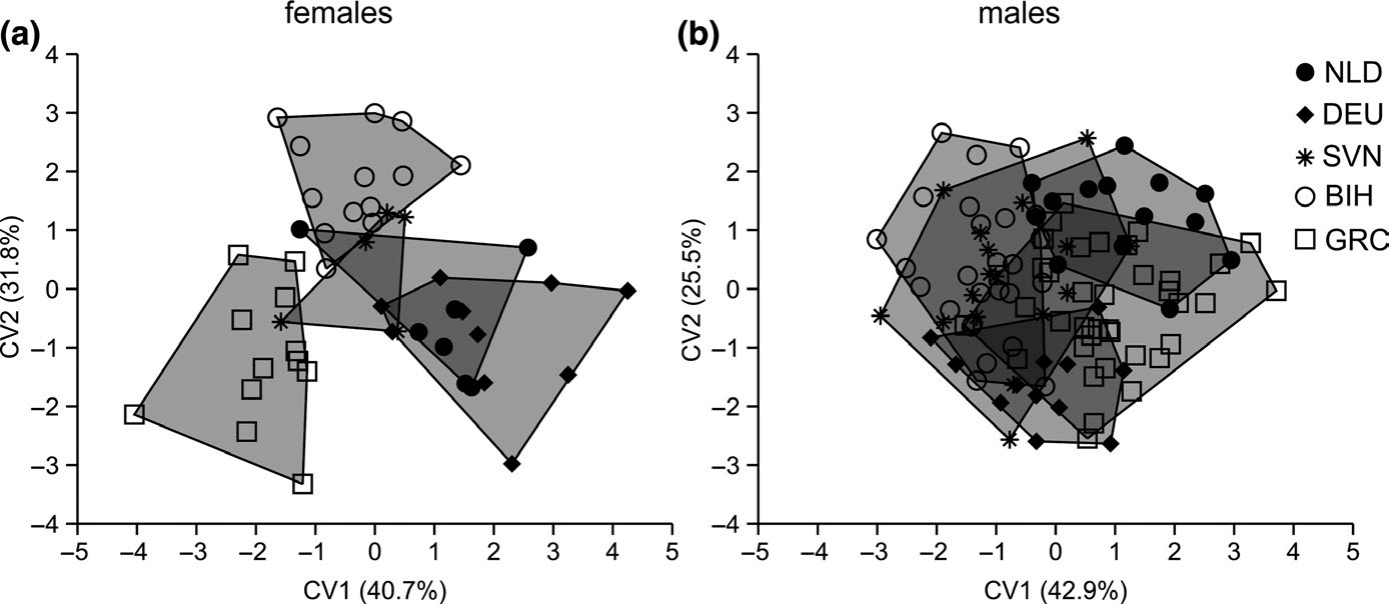
Scatterplot of individual scores from the CVA showing wing shape differentiation between the *Sphaerophoria scripta* populations: (a) females, (b) males. The percentage of explained variance of each CV is in parentheses. NLD—the Netherlands (the Hague), DEU—Germany (Berlin), SVN—Slovenia (Bled), BIH—Bosnia and Herzegovina (Banja Luka), GRC—Greece (Araxos)

**FIGURE 10:**
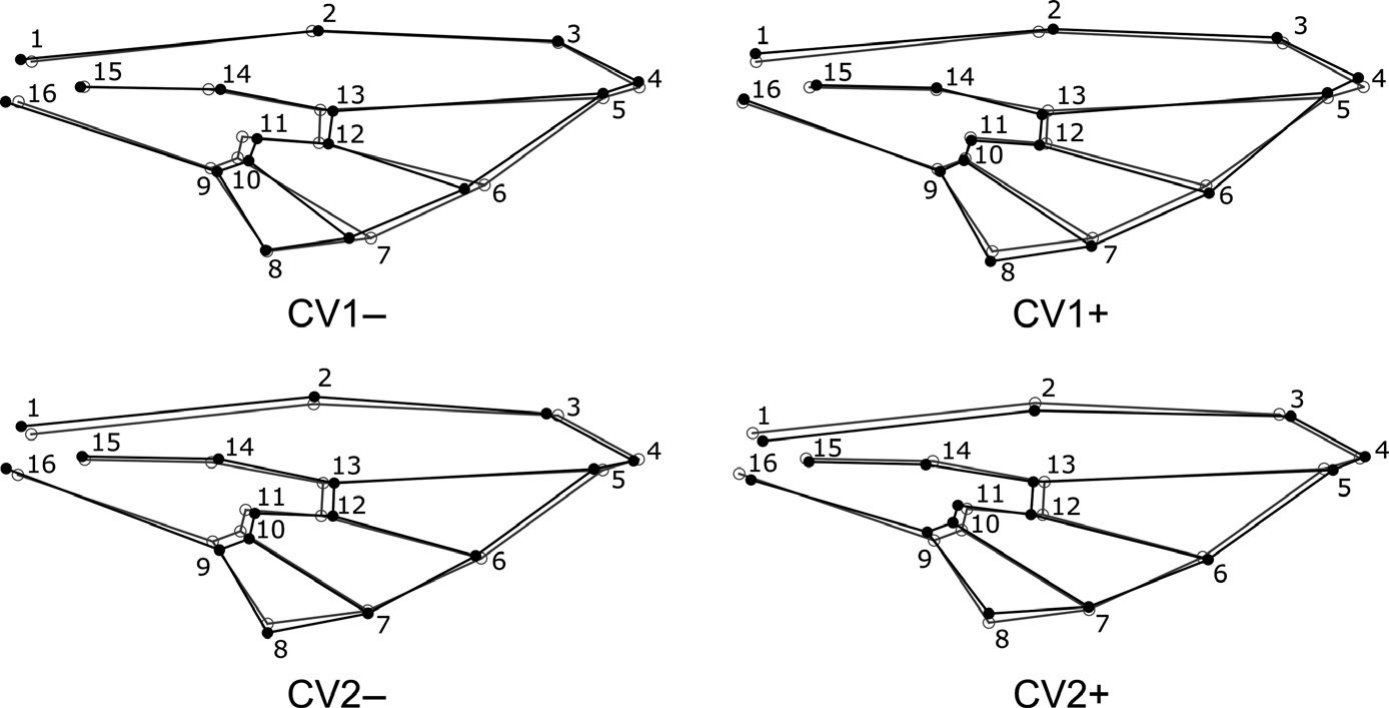
The wireframe graphs represent male wing shape changes (black lines) from consensus configuration of landmarks (gray lines) along the first two CVs. Numbers in the deformation grids refer to landmarks shown in Figure 3

Permutational MANOVA revealed that in females interpopulation differences in wing shape were not significant (*F* = 0.78, *p* = .84) despite populations being separated on CVA scatterplot (Figure 9). Moreover, pairwise Procrustes distances ranged from 0.0068 to 0.0110 and were nonsignificant (*p* > .05) for all population pairs (Table 3). Percentage of correct classification was 87.2% (10.6% jackknifed). Contrary to females, among males significant differences in wing shape were found (*F* = 2.11, *p* < .001). Procrustes distances ranged from 0.0072 to 0.0143 and were significant for five population pairs (Table 3). Furthermore, scatterplot obtained from CVA showed that along the first CV that accounting for 42.9% of total shape variation males from BIH partially separated from males from NLD, DEU, and GRC, while along CV2 (25.5%) males from NLD and GRC clustered with no overlap (Figure 9). Percentage of correct classification was 74.8% (29.0% jackknifed). Deformation grids showed that displacement of all landmarks contributed to shape differences between males of analyzed populations (Figure 10).

**TABLE 3:** Pairwise Procrustes distances between females (below diagonal) and males (above diagonal) of five *Sphaerophoria scripta* populations

	NLD	DEU	SVN	BIH	GRC
NLD	‐‐‐‐‐	0.0143***	0.0087	0.0110*	0.0083
DEU	0.0076	‐‐‐‐‐	0.0122*	0.0112*	0.0079
SVN	0.0110	0.0075	‐‐‐‐‐	0.0072	0.0083
BIH	0.0090	0.0068	0.0071	‐‐‐‐‐	0.0099**
GRC	0.0094	0.0083	0.0098	0.0094	‐‐‐‐‐

Note

Shaded boxes indicate significant pairwise comparison (***p < .001; **p < .01; and *p < .05).

## 4 DISCUSSION

### 4.1 The utility of the *COI* mtDNA in delimiting *Sphaerophoria* species

In this study, a mitochondrial, cytochrome *c* oxidase subunit I gene (*COI* mtDNA) was used to test its usefulness in determining the species borders within the *Sphaerophoria* genus of hoverflies. In particular, we utilized a partial, 481 bp‐long fragment of the standard, 658 bp‐long DNA barcoding marker (Folmer et al., 1994). The results of the analysis indicated that eight haplotypes (h1, h8, h16, h17, h18, h20, h27, and h32) were shared between two or more *Sphaerophoria* species (Table S5), with the consequent lowest interspecies *p* divergence of 0% rendering this barcode marker inadequate for the separation of different *Sphaerophoria* congeners. The inclusion of the longer fragments of the standard 5’ *COI* mtDNA (Table S2) did not improve the diagnostic value of 5’ *COI* mtDNA as a DNA barcode marker in *Sphaerophoria* genus. A similar conclusion was reached by Virgilio, Backeljau, Nevado, and De Meyer (2010), studying the barcode utility of different lengths and different regions of this marker across different insect orders, and finding that the applicability of the marker is not significantly affected by using its shorter fragments until the case of using 40 bp‐long mini‐barcodes. Apart from *COI* mtDNA used in this study, *18S* rRNA gene was previously used to decipher the phylogenetic relationships within Syrphidae family (Mengual, Ståhls, & Rojo, 2008). *Sphaerophoria scripta* and *S. philanthus* could not be separated apart using this slowly evolving marker, and neither could *S. contigua* from *S. macrogaster* as they shared identical haplotypes. These species pairs could however be distinguished from *S. rueppelii* and *S. loewi*, albeit by up to two substitutions (Mengual et al., 2008). Therefore, the future delimitation of different species of *Sphaerophoria* genus should incorporate a multi‐character approach, possibly using faster‐evolving loci than barcodes due to the supposed recent divergence of the studied taxa, as evidenced in the lack of the barcode gap between them.

The lack of the utility of 5’ *COI* mtDNA to species delimitation in *Sphaerophoria* genus has important implications for the first goal of this study—recognizing whether there was cryptic diversity within *S. scripta*. Namely, we retrieved two distinct haplogroups when analyzing the longer, 1,209 bp‐long fragment of this gene. Although no barcode gap separated the members of the major and minor haplogroups (Table S1), the members of the minor haplogroup were distinguished by a specific combination of six substitutions. However, this does not represent a strong evidence of putative cryptic diversity within *S. scripta* given the disputed resolution of this marker. Furthermore, *ITS2* rDNA alleles A and B were present among the members of both the major and minor haplogroups (Table S3). Therefore, rather than representing distinct taxa, the existence of two haplogroups oriented around two central haplotypes (H1 and H27) is indicative of the past population expansion from two previously separated sources (the dumbbell pattern of haplotype network, Avise, 2000). This is also evident in the geographical codistribution of the members of both haplogroups, and also in the fact that the less frequent *ITS2* rDNA allele B was retained in the representatives of the central haplotypes. Furthermore, our results are compatible with the conclusion of Morgan‐Richards et al. (2017) that large mitochondrial gene variability can be maintained in large populations due to the mutation‐drift equilibrium: there is an increased potential for new haplotypes to arise, but they are slow to be lost via genetic drift due to the large effective size. This is indeed supported by the conclusion of Raymond et al. (2013), where a large genetic diversity was also observed in *S. scripta* based on microsatellite loci as nuclear markers. Summarily, we conclude that *COI* mtDNA is an inadequate barcoding marker for the *Sphaerophoria* genus.

### 4.2 Population structure of *Sphaerophoria scripta* in Europe

The remaining study goals included the inspection of the population's structure of *S. scripta* based on an integrative approach combining two classes of molecular markers (phenotypic‐allozymes; and sequence‐based *COI* mtDNA) and morphological phenotypic characters of adult wings. We found that Bayesian clustering methods (BAPS, STRUCTURE, Geneland) indicated high gene flow among the five European geographic samples we analyzed, either by recognizing a single panmictic population within the total sample or by showing a high degree of admixture of several clusters for each of the sampling localities. This scenario illustrates a long‐standing problem in population genetics of highly migratory insects where deciphering the exact population structure is often challenging due to weak genetic differentiation among the analyzed localities and the resolution provided by traditional methods (see Liu, Chen, et al., 2019), however with the advent of next‐generation sequencing, novel approaches such as double digest Restriction‐site Associated DNA sequencing (ddRADseq) might be robust enough to resolve this issue (Liu, Chen, et al., 2019). This approach to resolving interpopulation relationships on a finer scale should be tested in the future for *S. scripta*; however, the aforementioned pattern of high genetic admixture, combined with Mantel's test—testing isolation by distance pattern, being nonsignificant, points toward a homogenous genetic structure in *S. scripta* within the assessed geographic transect. This finding is concordant with the study of Raymond et al. (2013), where a single panmictic unit was recognized across 12 European populations using microsatellite loci. Furthermore, population‐based analyses of genetic structure (AMOVA, pairwise Φ_ST_/*F*_ST_) also indicated high genetic connectedness of the assessed *S. scripta* populations, with Φ_ST_ being statistically nonsignificant in the case of *COI* mtDNA, while *F*_ST_ amounted only to 0.08 in the case of allozymes. Finally, testing for the significant differences between geographic samples based on variability of wing traits (size and shape) showed opposite results regarding the two sexes. Namely, no statistically significant differences were detected in either trait for females, while significant interpopulation differences were detected both in wing size and shape for males. This could point toward female‐biased dispersal (as no genetic differentiation was registered in *COI* mtDNA, a marker which is transmitted through the maternal line only), but it could also be indicative of the differences in sample size we analyzed (47 females vs. 107 males). The differences among males originating from different geographic locations in two wing morphometric characters which differ in their heritability (Bitner‐Mathé & Klaczko, 1999) could, apart from differential selective regimes they experience, indeed be indicative of the restricted dispersal of male specimens. However, this factor ultimately does not significantly contribute to the establishment of differentiated genetic clusters, as the homogenizing effect of the total gene flow was evident across the genomic loci which we analyzed.

### 4.3 Implications for conservation biological control

In the light of the current global climate changes, as well as human‐mediated habitat fragmentation, hoverflies represent a particularly noteworthy group of pest management interest as they possess a favorable configuration of biological traits which enables them rapid responses to changes in resource availability (Rader et al., 2020). Given that conserving enemy biodiversity has a strong impact on pest abundance in agricultural ecosystems (Jonsson et al., 2017), a precise identification of natural predators was integrated in pest management programs (Gomez‐Polo et al., 2014; Happe et al., 2019). We observed a lack of diagnostic value of *COI* mtDNA as a barcoding fragment in delimitation of closely related species of the genus *Sphaerophoria*, which implies the need for an integrated approach in assessing the biocontrol communities. Given that the use of a single mitochondrial gene failed to successfully delimit different *Sphaerophoria* species, the future attempts at their molecular identification should incorporate information from several genomic regions. Since sustainable land use and ecosystem services depend on diversity, identification, and conservation of key predators (Happe et al., 2019), assessing species borders of beneficial insects such as aphidophagous hoverflies would provide a solid base for sustainable biological control and pest management in agroecosystems (Happe et al., 2019; Le Hesran et al., 2019; Weintraub et al., 2017).

Finally, by using a combination of several molecular and phenotypic markers we showed the absence of population structuring of the long hoverfly *S. scripta* across the five European localities. Our integrative study confirms a great dispersal capacity of this migratory species, which is compatible with the results from previous study (Raymond et al., 2013), where a different marker class was used (microsatellite loci). Hence, population connectivity observed for one of the main aphid biocontrol agents on cereal crops and orchards (e.g., Chabert & Sarthou, 2017) would significantly contribute to the pest management programs. In addition, by combining genetic‐morphological approach we assessed a great evolutionary potential of this natural enemy species, which is crucial for maintaining sustainable ecosystem services under environmental changes (Le Hesran et al., 2019; Rader et al., 2020). Finally, connectivity among conspecific populations of the aphidophagous hoverflies across Europe enhances biological control of herbivorous pest insects which further provides sustainable ecosystem services. Unlike many beneficial species which are negatively affected by environmental fluctuations caused by intense agricultural practices, *S. scripta* showcases low genetic structuring on the one hand and high genetic diversity on the other hand. These traits are associated with higher probability for survival by providing high levels of standing genetic variability, by indicating a high tolerance to environmental variability, and by increasing colonization ability of species, which are highly favorable traits in the current context of global climate change and habitat fragmentation in agroecosystems (Liu, Xiaoqiang, et al., 2019; Raymond et al., 2013). Therefore, due to the specific traits of *S. scripta* (worldwide distribution, great genetic diversity, high phenotypic plasticity, intense gene flow, and population connectivity), this taxon represents a powerful agent of biological control. In the light of conservation biological control, it would therefore be of essential importance to protect its microhabitats within agroecosystems and local habitats that provide natural nurseries for natural enemy biodiversity (Gontijo, 2019; Snyder, 2019). For instance, it has been suggested that ungrazed patches in grazed grassland are valuable shelters for aphidophagous hoverflies (Speight, Good, & Sarthou, 2000). Likewise, cattle grazing and overall nitrogen enrichment of the soil represent effective strategies which could enhance the predator potential of *S. scripta* as plants which benefit from nitrogen‐rich soil are particularly beneficial to aphids, therefore providing prey to the long hoverfly and bolstering its population connectivity by providing suitable patches between agroecosystems (Chabert & Sarthou, 2017; Happe et al., 2019).

## CONFLICT OF INTEREST

The authors declare no conflict of interest.

## AUTHOR CONTRIBUTIONS

**Nemanja Gojković:** Data curation (equal); Formal analysis (lead); Software (lead); Visualization (equal); Writing‐original draft (equal); Writing‐review & editing (equal). **Ljubinka Francuski:** Conceptualization (equal); Data curation (equal); Investigation (equal); Methodology (lead); Supervision (equal); Writing‐review & editing (equal). **Jasmina Ludoški:** Data curation (equal); Formal analysis (equal); Methodology (equal); Software (equal); Visualization (equal); Writing‐original draft (equal); Writing‐review & editing (equal). **Vesna Milankov:** Conceptualization (equal); Data curation (equal); Funding acquisition (lead); Investigation (equal); Methodology (supporting); Project administration (lead); Resources (lead); Supervision (lead); Validation (equal); Writing‐original draft (equal); Writing‐review & editing (equal).

## Supporting information

Supplementary Material

Tables S1 and S2

## Data Availability

The DNA sequences analyzed in the manuscript have been archived in GenBank, while the other data supporting the results and conclusions were included in the additional files of the article.
