# Supplementary material for: DNA barcode assessment and population structure of aphidophagous hoverfly Sphaerophoria scripta: Implications for conservation biological control
Source: Ecol Evol. 2020 Aug 7;10(17):9428–43. doi: 10.1002/ece3.6631 (PMC7487226; doi:10.1002/ece3.6631)
Supplement: Supplementary file 1 — Supplementary Material [file ECE3-10-9428-s001.docx]

**DNA barcode assessment and population structure of aphidophagous hoverfly *Sphaerophoria scripta*:**

**Implications for conservation biological control**

| **Supp. Table S1.** The list of sequences downloaded from BOLD and GenBank (sequence accession codes given in italic) databases and used in the analysis of the barcode utility of 5’ *COI* mtDNA for the delimitation of *Sphaerophoria* species | |
| --- | --- |
| *Sphaerophoria* species | BOLD/*GenBank* accession code |
| *S. scripta* | BBDCN791-10, BBDEC354-09, BEEEE421-16, CGDTA056-09, CGDTA057-09, CGDTA065-09, CNCDB3328-11, GBDP5732-09, GBSYR143-14, GBSYR144-14, GBSYR145-14, GBSYR146-14, *KC900480*, *KR260241*, NORSY019-12, NORSY053-12, NORSY217-12, ZMUCG014-12, ZMUCG015-12, ZMUCG016-12 |
| *S. philanthus* | CNGBJ1873-14, CNGBJ1911-14, CNGBJ1916-14, CNGBJ1918-14, CNJAC1175-12, CNJAD2391-12, CNJAG1182-12, CNJAG1188-12, CNJAG1210-12, CNJAG1211-12, CNJAG1228-12, JSFLA011-08, JWDCE918-10, JWDCE919-10, JWDCG052-10, JWDCG053-10, JWDCH675-10, JWDCH819-10, JWDCI418-10, JWDCJ1402-11, JWDCJ1404-11, JWDCJ1406-11, JWDCJ1484-11, JWDCJ733-11, JWDCJ850-11, JWDCK125-11, JWDCK236-11, JWDCK239-11, JWDCK240-11, JWDCL247-11, JWDCL407-11, *KC900490*, LRSYR004-07, LRSYR015-07, LRSYR030-07, LRSYR035-07, LRSYR036-07, LRSYR038-07, LRSYR039-07, LRSYR045-07, MHFLY127-07, MHSYR028-06, MHSYR059-07, MHSYR060-07, MHSYR098-07, MHSYR100-07, MHSYR101-07, MHSYR102-07, MHSYR105-07, MHSYR108-07, MHSYR111-07, MHSYR222-07, MHSYR296-07, MHSYR297-07, MHSYR298-07, MHSYR300-07, MHSYR301-07, MHSYR302-07, MHSYR303-07, NORSY030-12, NORSY081-12, NORSY252-12, TTDFW243-08, TTDFW342-08, TTDFW363-08, TTDFW458-08, TTDFW659-08, TWDIP022-09, TWDIP023-09, TWDIP024-09, TWDIP067-09, UAMIC2960-15, UAMIC3018-15 |
| *S. contigua* | CNJAA146-12, CNJAB1073-12, CNJAB1087-12, CNJAC1080-12, CNJAC1083-12, CNJAC1384-12, CNJAC1622-12, CNJAC1628-12, CNJAC1629-12, CNJAC1659-12, CNJAC1664-12, CNJAC1677-12, CNJAC1683-12, CNJAC1686-12, CNJAC1693-12, CNJAC1697-12, CNJAC1699-12, CNJAD2402-12, CNJAD2410-12, CNJAD2450-12, CNJAD2457-12, CNJAD2469-12, CNMID1044-14, CNMIF047-14, CNPKF1647-14, CNPKF1648-14, CNPKF1653-14, CNPKF1676-14, GBDP5734-09, *KC900440*, TTDBW089-08, TTDBW754-09, TTDBW916-09, TTDFW246-08, TTDFW249-08, TTDFW663-08, TTDFW743-08, TTDFW744-08, TTDFW745-08, TTDFW794-08, TTDFW798-08, TTDFW802-08, TTDFW803-08, TTDFW805-08, TTDFW806-08, TTDFW807-08, TTDFW809-08, TTDFW902-08, TTDFW903-08, TTDFW973-08 |
| *S. novaeangliae* | ASDIP282-15, ASDIP285-15, CNCDB3240-11, CNFNE002-14, CNFNE033-14, CNFNE039-14, CNFNE044-14, CNFNE047-14, CNFNE069-14, CNFNE076-14, CNFNE079-14, CNFNE1828-14, CNFNE1834-14, CNFNE1835-14, CNFNE1848-14, CNFNE2999-14, CNFNF024-14, CNFNF036-14, CNFNF057-14, CNFNF1740-14,  CNFNF1753-14, CNFNF1775-14, CNFNF1795-14, CNFNG204-14, CNFNH024-14, CNFNQ625-14, CNFNR1023-14, CNFNR1962-14, CNFNR1968-14, CNFNR228-14, CNFNR3006-14, CNFNR3029-14, CNFNR3037-14, CNFNR3046-14, CNFNR3059-14, CNFNS026-14, CNFNT1991-14, CNFNU022-14, CNRME2424-12, *KC900486* |
| *S. abbreviata* | CNFNE2866-14, CNFNQ611-14, CNFNQ630-14, CNFNR3038-14, CNMIG748-14, CNMIM109-14 |
| *S. rueppellii* | GBSYR141-14, GBSYR142-14, NORSY384-12, TTDFW407-08 |
| *S. sulphuripes* | BBDIV514-12, SMTPI6980-14, SMTPI6989-14, SSDWA7337-15 |
| *S. shirchan* | GMGRB1536-13, GMGRB1537-13, GMGRC2641-13 |
| *S. taeniata* | NORSY009-12, NORSY114-12, NORSY214-12 |
| *S. virgata* | NORSY112-12, NORSY142-12, NORSY274-12 |
| *S. bankowskae* | NORSY495-15, NORSY496-15 |
| *S. macrogaster* | GBMIN62181-17, GBMIN62182-17 |
| *S. interrupta* | NORSY117-12, NORSY231-12 |
| *S. batava* | NORSY047-12, NORSY160-12 |
| *S. laurae* | NORSY416-12, NORSY421-12 |
| *S. pyrrhina* | CNCDB3322-11 |
| *S. fatarum* | BEEEE415-16 |
| *S. bengalensis* | CGDTA055-09 |
| *S. longipilosa* | CNGBJ2023-14 |
| *S. asymmetrica* | *KC900483* |

| **Supp. Table S2.** The data regarding the full length of the sequences belonging to different *Sphaerophoria* species which represented identical respective haplotypes in the utility analysis of the 481 bp-long fragment of 5’ *COI* mtDNA as a DNA barcode marker in *Sphaerophoria* genus. The missing data content refers to the standard, 658 bp-long fragment of 5’ *COI* mtDNA (Folmer et al., 1994). Variable positions were defined relative to their positions in the mitochondrial genome of *Episyrphus balteatus* (GenBank accession code: KU351241). Shaded boxes indicate allospecific individuals representing identical respective haplotypes despite being fully sequenced in the standard barcode region. Taxon abbreviations include the following *Sphaerophoria* species: AS – *S. asymmetrica*, BA – *S. bankowskae*, BE – *S. bengalensis*, CO – *S. contigua*, FA – *S. fatarum*, IN – *S. interrupta*, LA – *S. laurae*, PH – *S. philanthus*, SC – *S. scripta*, RU – *S. rueppellii*, TA – *S. taeniata*, and VI – *S. virgata* | | | | | | | | | |
| --- | --- | --- | --- | --- | --- | --- | --- | --- | --- |
| BOLD/*GenBank* Accession code | Taxon | Missing 5’ *COI* mtDNA barcode content [bp] | | | Variable positions within the barcode fragment separating previously identical sequences | | | | |
|  |  | 5' end | 3' end | Total | **1546** | **2155** | **2161** | **2176** | **2179** |
| **h1** |  |  |  |  | **A** | **C** | **C** | **A** | **C** |
| BBDCN791-10 | SC | 0 | 100 | 100 | **.** | **.** | **.** | **.** | **.** |
| BBDEC354-09 | SC | 0 | 0 | 0 | **.** | **.** | **.** | **.** | **.** |
| BEEEE421-16 | SC | 0 | 54 | 54 | **.** | **.** | **.** | **.** | **.** |
| CGDTA056-09 | SC | 0 | 0 | 0 | **.** | **.** | **.** | **.** | **.** |
| GBDP5732-09 | SC | 16 | 0 | 16 | **.** | **.** | **.** | **.** | **.** |
| GBSYR143-14 | SC | 45 | 38 | 83 | **.** | **.** | **.** | **.** | **.** |
| GBSYR144-14 | SC | 33 | 40 | 73 | **.** | **.** | **.** | **.** | **.** |
| GBSYR145-14 | SC | 44 | 38 | 82 | **.** | **.** | **.** | **.** | **.** |
| GBSYR146-14 | SC | 51 | 40 | 91 | **.** | **.** | **.** | **.** | **.** |
| NORSY019-12 | SC | 0 | 0 | 0 | **.** | **.** | **.** | **.** | **.** |
| NORSY217-12 | SC | 0 | 0 | 0 | **.** | **.** | **.** | **.** | **.** |
| NS545 | SC | 36 | 0 | 36 | **.** | **.** | **.** | **.** | **.** |
| NS546 | SC | 36 | 0 | 36 | **.** | **.** | **.** | **.** | **.** |
| NS548 | SC | 36 | 0 | 36 | **.** | **.** | **.** | **.** | **.** |
| NS549 | SC | 36 | 0 | 36 | **.** | **.** | **.** | **.** | **.** |
| NS550 | SC | 36 | 0 | 36 | **.** | **.** | **.** | **.** | **.** |
| NS554 | SC | 36 | 0 | 36 | **.** | **.** | **.** | **.** | **.** |
| NS555 | SC | 36 | 0 | 36 | **.** | **.** | **.** | **.** | **.** |
| NS556 | SC | 36 | 0 | 36 | **.** | **.** | **.** | **.** | **.** |
| NS1086 | SC | 38 | 0 | 38 | **.** | **.** | **.** | **.** | **.** |
| NS1088 | SC | 36 | 0 | 36 | **.** | **.** | **.** | **.** | **.** |
| NS1089 | SC | 47 | 0 | 47 | **.** | **.** | **.** | **.** | **.** |
| NS1090 | SC | 36 | 0 | 36 | **.** | **.** | **.** | **.** | **.** |
| NS1092 | SC | 36 | 0 | 36 | **.** | **.** | **.** | **.** | **.** |
| NS1093 | SC | 36 | 0 | 36 | **.** | **.** | **.** | **.** | **.** |
| NS1096 | SC | 36 | 0 | 36 | **.** | **.** | **.** | **.** | **.** |
| NS1098 | SC | 36 | 0 | 36 | **.** | **.** | **.** | **.** | **.** |
| NS1101 | SC | 36 | 0 | 36 | **.** | **.** | **.** | **.** | **.** |
| NS1102 | SC | 65 | 0 | 65 | **.** | **.** | **.** | **.** | **.** |
| NS1103 | SC | 36 | 0 | 36 | **.** | **.** | **.** | **.** | **.** |
| NS1104 | SC | 36 | 0 | 36 | **.** | **.** | **.** | **.** | **.** |
| NS1105 | SC | 36 | 0 | 36 | **.** | **.** | **.** | **.** | **.** |
| NS1106 | SC | 36 | 0 | 36 | **.** | **.** | **.** | **.** | **.** |
| NS1107 | SC | 36 | 0 | 36 | **.** | **.** | **.** | **.** | **.** |
| NS1109 | SC | 36 | 0 | 36 | **.** | **.** | **.** | **.** | **.** |
| NS1110 | SC | 36 | 0 | 36 | **.** | **.** | **.** | **.** | **.** |
| NS1253 | SC | 36 | 0 | 36 | **.** | **.** | **.** | **.** | **.** |
| NS1257 | SC | 53 | 0 | 53 | **.** | **.** | **.** | **.** | **.** |
| NS1263 | SC | 39 | 0 | 39 | **.** | **.** | **.** | **.** | **.** |
| NS1268 | SC | 64 | 0 | 64 | **.** | **.** | **.** | **.** | **.** |
| NS1270 | SC | 36 | 0 | 36 | **.** | **.** | **.** | **.** | **.** |
| NS1273 | SC | 41 | 0 | 41 | **.** | **.** | **.** | **.** | **.** |
| NS1275 | SC | 63 | 0 | 63 | **.** | **.** | **.** | **.** | **.** |
| NS1277 | SC | 63 | 0 | 63 | **.** | **.** | **.** | **.** | **.** |
| NS1281 | SC | 65 | 0 | 65 | **.** | **.** | **.** | **.** | **.** |
| NS1286 | SC | 36 | 0 | 36 | **.** | **.** | **.** | **.** | **.** |
| NS1267 | SC | 49 | 0 | 49 | **.** | **.** | **.** | **.** | **.** |
| NS1272 | SC | 36 | 0 | 36 | **.** | **.** | **.** | **.** | **.** |
| *KC900480* | SC | 0 | 0 | 0 | **.** | **.** | **.** | **.** | **.** |
| *KR260241* | SC | 1 | 7 | 8 | **.** | **.** | **.** | **.** | **.** |
| LRSYR036-07 | PH | 0 | 0 | 0 | **.** | **.** | **.** | **.** | **.** |
| MHSYR060-07 | PH | 0 | 13 | 13 | **.** | **.** | **.** | **.** | **.** |
| MHSYR297-07 | PH | 0 | 0 | 0 | **.** | **.** | **.** | **.** | **.** |
| MHSYR298-07 | PH | 38 | 27 | 65 | **.** | **.** | **.** | **.** | **.** |
| UAMIC3018-15 | PH | 0 | 0 | 0 | **.** | **.** | **.** | **.** | **.** |
| CGDTA055-09 | BE | 0 | 40 | 40 | **.** | **.** | **.** | **.** | **.** |
| NS1095 | SC | 36 | 0 | 36 | **.** | **T** | **.** | **.** | **.** |
| NS1254 | SC | 42 | 0 | 42 | **.** | **.** | **T** | **.** | **.** |
| NS1284 | SC | 51 | 0 | 51 | **.** | **.** | **T** | **.** | **.** |
| JWDCE919-10 | PH | 0 | 0 | 0 | **.** | **.** | **.** | **.** | **T** |
| JWDCG052-10 | PH | 0 | 0 | 0 | **.** | **.** | **.** | **.** | **T** |
| JWDCH819-10 | PH | 0 | 0 | 0 | **.** | **.** | **.** | **.** | **T** |
| JWDCJ1406-11 | PH | 0 | 0 | 0 | **.** | **.** | **.** | **.** | **T** |
| JWDCJ850-11 | PH | 0 | 0 | 0 | **.** | **.** | **.** | **.** | **T** |
| JWDCK125-11 | PH | 0 | 0 | 0 | **.** | **.** | **.** | **.** | **T** |
| JWDCK239-11 | PH | 0 | 0 | 0 | **.** | **.** | **.** | **.** | **T** |
| JWDCL407-11 | PH | 0 | 0 | 0 | **.** | **.** | **.** | **.** | **T** |
| LRSYR004-07 | PH | 0 | 0 | 0 | **.** | **.** | **.** | **.** | **T** |
| LRSYR015-07 | PH | 0 | 0 | 0 | **.** | **.** | **.** | **.** | **T** |
| MHSYR102-07 | PH | 2 | 0 | 2 | **.** | **.** | **.** | **.** | **T** |
| MHSYR108-07 | PH | 0 | 0 | 0 | **.** | **.** | **.** | **.** | **T** |
| MHSYR111-07 | PH | 0 | 0 | 0 | **.** | **.** | **.** | **.** | **T** |
| TTDFW342-08 | PH | 0 | 0 | 0 | **.** | **.** | **.** | **.** | **T** |
| TTDFW363-08 | PH | 0 | 0 | 0 | **.** | **.** | **.** | **.** | **T** |
| TTDFW458-08 | PH | 0 | 0 | 0 | **.** | **.** | **.** | **.** | **T** |
| *KC900490* | PH | 0 | 0 | 0 | **.** | **.** | **.** | **.** | **T** |
| MHSYR101-07 | PH | 0 | 0 | 0 | **G** | **.** | **.** | **.** | **T** |
| **h8** |  |  |  |  | **A** | **C** | **C** | **A** | **C** |
| NS553 | SC | 36 | 0 | 36 | **.** | **.** | **.** | **.** | **.** |
| NS1094 | SC | 45 | 0 | 45 | **.** | **.** | **.** | **.** | **.** |
| NS1271 | SC | 36 | 0 | 36 | **.** | **.** | **.** | **.** | **.** |
| TTDFW659-08 | PH | 0 | 0 | 0 | **.** | **.** | **.** | **.** | **T** |
| **h16** |  |  |  |  | **A** | **C** | **C** | **A** | **C** |
| JSFLA011-08 | PH | 0 | 0 | 0 | **.** | **.** | **.** | **.** | **.** |
| JWDCG053-10 | PH | 0 | 0 | 0 | **.** | **.** | **.** | **.** | **.** |
| JWDCJ1402-11 | PH | 0 | 0 | 0 | **.** | **.** | **.** | **.** | **.** |
| JWDCJ1484-11 | PH | 0 | 0 | 0 | **.** | **.** | **.** | **.** | **.** |
| JWDCK236-11 | PH | 0 | 0 | 0 | **.** | **.** | **.** | **.** | **.** |
| LRSYR030-07 | PH | 0 | 0 | 0 | **.** | **.** | **.** | **.** | **.** |
| LRSYR045-07 | PH | 0 | 0 | 0 | **.** | **.** | **.** | **.** | **.** |
| MHSYR059-07 | PH | 0 | 0 | 0 | **.** | **.** | **.** | **.** | **.** |
| MHSYR100-07 | PH | 0 | 0 | 0 | **.** | **.** | **.** | **.** | **.** |
| MHSYR222-07 | PH | 0 | 0 | 0 | **.** | **.** | **.** | **.** | **.** |
| CNCDB3328-11 | SC | 0 | 0 | 0 | **.** | **.** | **.** | **.** | **.** |
| NS1289 | SC | 46 | 0 | 46 | **.** | **.** | **T** | **.** | **.** |
| JWDCI418-10 | PH | 0 | 0 | 0 | **.** | **.** | **.** | **.** | **T** |
| MHSYR302-07 | PH | 0 | 0 | 0 | **.** | **.** | **.** | **.** | **T** |
| MHSYR028-06 | PH | 5 | 3 | 8 | **.** | **.** | **T** | **.** | **T** |
| NORSY030-12 | PH | 0 | 0 | 0 | **.** | **.** | **.** | **T** | **T** |
| NORSY081-12 | PH | 0 | 0 | 0 | **.** | **.** | **.** | **T** | **T** |
| NORSY252-12 | PH | 0 | 0 | 0 | **.** | **.** | **.** | **T** | **T** |
| **h17** |  |  |  |  | **A** | **C** | **T** | **A** | **C** |
| GBSYR141-14 | RU | 46 | 37 | 83 | **.** | **.** | **.** | **.** | **.** |
| GBSYR142-14 | RU | 54 | 38 | 92 | **.** | **.** | **.** | **.** | **.** |
| NORSY384-12 | RU | 0 | 0 | 0 | **.** | **.** | **.** | **.** | **.** |
| CGDTA065-09 | SC | 0 | 0 | 0 | **.** | **.** | **.** | **.** | **.** |
| **h18** |  |  |  |  | **A** | **C** | **C** | **A** | **T** |
| GBDP5734-09 | CO | 28 | 0 | 28 | **.** | **.** | **.** | **.** | **.** |
| TTDBW089-08 | CO | 0 | 24 | 24 | **.** | **.** | **.** | **.** | **.** |
| TTDFW902-08 | CO | 0 | 0 | 0 | **.** | **.** | **.** | **.** | **.** |
| TTDFW903-08 | CO | 0 | 0 | 0 | **.** | **.** | **.** | **.** | **.** |
| TTDFW973-08 | CO | 0 | 17 | 17 | **.** | **.** | **.** | **.** | **.** |
| *KC900440* | CO | 0 | 0 | 0 | **.** | **.** | **.** | **.** | **.** |
| TTDBW916-09 | CO | 43 | 77 | 120 | **.** | **.** | **.** | **.** | **.** |
| JWDCJ1404-11 | PH | 0 | 0 | 0 | **.** | **.** | **.** | **.** | **.** |
| BEEEE415-16 | FA | 0 | 54 | 54 | **.** | **.** | **.** | **.** | **.** |
| NORSY496-15 | BA | 0 | 0 | 0 | **.** | **.** | **.** | **.** | **.** |
| *KC900483* | AS | 0 | 0 | 0 | **.** | **.** | **.** | **.** | **.** |
| UAMIC2960-15 | PH | 0 | 0 | 0 | **.** | **.** | **T** | **.** | **.** |
| **h20** |  |  |  |  | **A** | **C** | **T** | **A** | **T** |
| TTDBW754-09 | CO | 0 | 0 | 0 | **.** | **.** | **.** | **.** | **.** |
| TTDFW246-08 | CO | 0 | 0 | 0 | **.** | **.** | **.** | **.** | **.** |
| TTDFW249-08 | CO | 0 | 0 | 0 | **.** | **.** | **.** | **.** | **.** |
| TTDFW663-08 | CO | 0 | 0 | 0 | **.** | **.** | **.** | **.** | **.** |
| TTDFW743-08 | CO | 0 | 0 | 0 | **.** | **.** | **.** | **.** | **.** |
| TTDFW744-08 | CO | 0 | 0 | 0 | **.** | **.** | **.** | **.** | **.** |
| TTDFW745-08 | CO | 0 | 0 | 0 | **.** | **.** | **.** | **.** | **.** |
| TTDFW794-08 | CO | 0 | 0 | 0 | **.** | **.** | **.** | **.** | **.** |
| TTDFW798-08 | CO | 0 | 0 | 0 | **.** | **.** | **.** | **.** | **.** |
| TTDFW802-08 | CO | 0 | 0 | 0 | **.** | **.** | **.** | **.** | **.** |
| TTDFW803-08 | CO | 0 | 0 | 0 | **.** | **.** | **.** | **.** | **.** |
| TTDFW805-08 | CO | 0 | 0 | 0 | **.** | **.** | **.** | **.** | **.** |
| TTDFW806-08 | CO | 0 | 0 | 0 | **.** | **.** | **.** | **.** | **.** |
| TTDFW807-08 | CO | 0 | 0 | 0 | **.** | **.** | **.** | **.** | **.** |
| TTDFW809-08 | CO | 0 | 0 | 0 | **.** | **.** | **.** | **.** | **.** |
| CNGBJ1873-14 | PH | 9 | 86 | 95 | **.** | **.** | **.** | **.** | **.** |
| CNGBJ1911-14 | PH | 0 | 79 | 79 | **.** | **.** | **.** | **.** | **.** |
| CNGBJ1916-14 | PH | 9 | 67 | 76 | **.** | **.** | **.** | **.** | **.** |
| CNJAC1175-12 | PH | 0 | 28 | 28 | **.** | **.** | **.** | **.** | **.** |
| CNJAD2391-12 | PH | 0 | 27 | 27 | **.** | **.** | **.** | **.** | **.** |
| CNJAG1182-12 | PH | 0 | 44 | 44 | **.** | **.** | **.** | **.** | **.** |
| CNJAG1188-12 | PH | 0 | 44 | 44 | **.** | **.** | **.** | **.** | **.** |
| CNJAG1210-12 | PH | 0 | 43 | 43 | **.** | **.** | **.** | **.** | **.** |
| CNJAG1211-12 | PH | 0 | 44 | 44 | **.** | **.** | **.** | **.** | **.** |
| CNJAG1228-12 | PH | 0 | 26 | 26 | **.** | **.** | **.** | **.** | **.** |
| TTDFW243-08 | PH | 0 | 0 | 0 | **.** | **.** | **.** | **.** | **.** |
| TTDFW407-08 | RU | 46 | 47 | 93 | **.** | **.** | **.** | **.** | **.** |
| NORSY495-15 | BA | 0 | 47 | 47 | **.** | **T** | **.** | **.** | **.** |
| **h27** |  |  |  |  | **A** | **C** | **C** | **A** | **C** |
| NORSY114-12 | TA | 0 | 0 | 0 | **.** | **.** | **.** | **.** | **.** |
| NORSY231-12 | IN | 0 | 0 | 0 | **.** | **.** | **.** | **.** | **.** |
| **h32** |  |  |  |  | **A** | **C** | **C** | **A** | **T** |
| NORSY112-12 | VI | 0 | 0 | 0 | **.** | **.** | **.** | **.** | **.** |
| NORSY142-12 | VI | 0 | 0 | 0 | **.** | **.** | **.** | **.** | **.** |
| NORSY274-12 | VI | 0 | 0 | 0 | **.** | **.** | **.** | **.** | **.** |
| NORSY416-12 | LA | 0 | 0 | 0 | **.** | **.** | **.** | **.** | **.** |

| **Supp. Table S3.** The details of the *Sphaerophoria scripta* individuals from this study which were *de novo* genotyped at *COI* mtDNA and *ITS2* rDNA loci. The individuals representing the less frequent allele B are shaded. NLD - the Netherlands (the Hague), DEU - Germany (Berlin), SVN - Slovenia (Bled), BIH - Bosnia and Herzegovina (Banja Luka), GRC - Greece (Araxos) | | | | | |
| --- | --- | --- | --- | --- | --- |
| Population | ID | *Sphaerophoria scripta*  genetic analysis  *COI* haplotypes (1209 bp) | *Sphaerophoria* genus  barcode utility  *COI* haplotypes (481 bp) | GenBank  accession code | *ITS2* alleles (426 bp) |
| NLD | NS545 | H1 | h1 |  | / |
|  | NS546 | H1 | h1 |  | A |
|  | NS1086 | H3 | h1 |  | A |
|  | NS1087 | H31 | h11 |  | A |
|  | NS1088 | H1 | h1 |  | A |
|  | NS1089 | H1 | h1 |  | A |
|  | NS1090 | H1 | h1 |  | A |
|  | NS1250 | H18 | h5 |  | A |
|  | NS1251 | H1 | / |  | A |
|  | NS1252 | / | / | / | A |
|  | NS1253 | H3 | h1 |  | / |
|  | NS1254 | H25 | h1 |  | / |
|  | NS1272 | / | h1 |  | / |
|  | NS1273 | H2 | h1 |  | / |
|  | NS1274 | H2 | / |  | / |
| DEU | NS547 | H1 | h1 |  | A |
|  | NS548 | H13 | h1 |  | A |
|  | NS1096 | H1 | h1 |  | B |
|  | NS1098 | H15 | h1 |  | / |
|  | NS1099 | H17 | h4 |  | A |
|  | NS1100 | H24 | h9 |  | A |
|  | NS1264 | H1 | / |  | A |
|  | NS1265 | / | / | / | A |
|  | NS1266 | H8 | / |  | A |
|  | NS1267 | / | h1 |  | / |
|  | NS1283 | H1 | / |  | / |
|  | NS1284 | H22 | h1 |  | / |
|  | NS1285 | H10 | / |  | / |
| SVN | NS549 | H1 | h1 |  | / |
|  | NS550 | H23 | h1 |  | A |
|  | NS1091 | H30 | h14 |  | A |
|  | NS1092 | H5 | h1 |  | A |
|  | NS1093 | H1 | h1 |  | A |
|  | NS1094 | H4 | h8 |  | A |
|  | NS1095 | H21 | h1 |  | A |
|  | NS1255 | H9 | / |  | A |
|  | NS1256 | H16 | h6 |  | / |
|  | NS1257 | H1 | h1 |  | / |
|  | NS1275 | H2 | h1 |  | / |
|  | NS1277 | H2 | h1 |  | / |
|  | NS1278 | H32 | h11 |  | / |
| BIH | NS553 | H4 | h8 |  | / |
|  | NS554 | H1 | h1 |  | A |
|  | NS1106 | H1 | h1 |  | B |
|  | NS1107 | H3 | h1 |  | / |
|  | NS1108 | H29 | h13 |  | A |
|  | NS1109 | H1 | h1 |  | B |
|  | NS1110 | H1 | h1 |  | A |
|  | NS1268 | H5 | h1 |  | A |
|  | NS1269 | H19 | h7 |  | / |
|  | NS1270 | H1 | h1 |  | / |
|  | NS1271 | / | h8 |  | / |
|  | NS1286 | H1 | h1 |  | A |
|  | NS1287 | H28 | h15 |  | / |
|  | NS1288 | H6 | h3 |  | / |
|  | NS1289 | H20 | h16 |  | / |
| GRC | NS555 | H1 | h1 |  | A |
|  | NS556 | H1 | h1 |  | A |
|  | NS1101 | H1 | h1 |  | / |
|  | NS1102 | H11 | h1 |  | A |
|  | NS1103 | H12 | h1 |  | A |
|  | NS1104 | H1 | h1 |  | A |
|  | NS1105 | H1 | h1 |  | / |
|  | NS1259 | H27 | / |  | B |
|  | NS1260 | H2 | / |  | / |
|  | NS1261 | H26 | / |  | A |
|  | NS1262 | H7 | / |  | / |
|  | NS1263 | H1 | h1 |  | / |
|  | NS1279 | H33 | h11 |  | A |
|  | NS1280 | H14 | / |  | / |
|  | NS1281 | H2 | h1 |  | / |
| TOTAL: |  | 66 | 57 |  | 39 |

| **Supp. Table S4.** The frequency of allozyme loci alleles registered in the five analyzed *Sphaerophoria scripta* populations and obtained from Genepop software. NLD - the Netherlands (the Hague), DEU - Germany (Berlin), SVN - Slovenia (Bled), BIH - Bosnia and Herzegovina (Banja Luka), GRC - Greece (Araxos) | | | | | |
| --- | --- | --- | --- | --- | --- |
| **Alleles** | **NLD** | **DEU** | **SVN** | **BIH** | **GRC** |
| ***Idh-1*** |  |  |  |  |  |
| *a* | 0.556 | 0.533 | 0.850 | 0.778 | 0.909 |
| *b* | 0.444 | 0.400 | 0.100 | 0.167 | 0.091 |
| *c* | 0.000 | 0.067 | 0.050 | 0.056 | 0.000 |
| ***Mdh-2*** |  |  |  |  |  |
| *a* | 0.000 | 0.300 | 0.667 | 0.300 | 0.167 |
| *b* | 0.917 | 0.600 | 0.333 | 0.600 | 0.278 |
| *c* | 0.083 | 0.100 | 0.000 | 0.100 | 0.556 |
| ***Me*** |  |  |  |  |  |
| *a* | 1.000 | 0.333 | 0.000 | 1.000 | 0.150 |
| *b* | 0.000 | 0.333 | 0.700 | 0.000 | 0.400 |
| *c* | 0.000 | 0.333 | 0.300 | 0.000 | 0.450 |
| ***Ao*** |  |  |  |  |  |
| *a* | 0.133 | 0.333 | 0.200 | 0.000 | 0.231 |
| *b* | 0.600 | 0.222 | 0.300 | 0.750 | 0.615 |
| *c* | 0.267 | 0.444 | 0.300 | 0.250 | 0.077 |
| *d* | 0.000 | 0.000 | 0.200 | 0.000 | 0.077 |
| Sample size | 18 | 15 | 21 | 18 | 22 |

| **Supp. Table S5.** The list of 5’ *COI* mtDNA haplotypes (481-bp long) used for the analysis of the marker’s barcode utility in *Sphaerophoria* genus, with details on the number of individuals from each analyzed *Sphaerophoria* species representing each haplotype. Haplotypes shared between two or more species are bolded. N_seq_- the total number of retrieved sequences per each haplotype | | |
| --- | --- | --- |
| Haplotype | N_seq_ | *Sphaerophoria* species |
| **h1** | 77 | 53 x *S.* *scripta*, 23 x *S.* *philanthus*, 1 x *S.* *bengalensis* |
| h2 | 1 | *S. scripta* |
| h3 | 1 | *S. scripta* |
| h4 | 1 | *S. scripta* |
| h5 | 1 | *S. scripta* |
| h6 | 1 | *S. scripta* |
| h7 | 1 | *S. scripta* |
| **h8** | 4 | 3 x *S.* *scripta*, 1 x *S. philanthus* |
| h9 | 1 | *S. scripta* |
| h10 | 1 | *S. scripta* |
| h11 | 6 | *S. scripta* |
| h12 | 1 | *S. scripta* |
| h13 | 1 | *S. scripta* |
| h14 | 1 | *S. scripta* |
| h15 | 1 | *S. scripta* |
| **h16** | 18 | *16 x S. philanthus*, *2 x S. scripta* |
| **h17** | 4 | 3 x *S.* *rueppellii*, 1 x *S. scripta* |
| **h18** | 12 | 7 x *S. contigua*, 2 x *S. philanthus*, 1 x *S. fatarum*, 1 x *S. bankowskae*, 1 x *S. asymmetrica* |
| h19 | 13 | *S. philanthus* |
| **h20** | 28 | 15 x *S. contigua*, 11 x *S. philanthus*, 1 x *S. bankowskae*, 1 x *S.* *rueppellii* |
| h21 | 1 | *S. philanthus* |
| h22 | 1 | *S. philanthus* |
| h23 | 2 | *S. philanthus* |
| h24 | 1 | *S. philanthus* |
| h25 | 2 | *S. philanthus* |
| h26 | 1 | *S. longipilosa* |
| **h27** | 2 | *S. taeniata*, *S. interrupta* |
| h28 | 1 | *S. interrupta* |
| h29 | 2 | *S. taeniata* |
| h30 | 1 | *S. batava* |
| h31 | 2 | *S. macrogaster* |
| **h32** | 4 | 3 x *S. virgata*, 1 x *S. laurae* |
| h33 | 1 | *S. laurae* |
| h34 | 1 | *S. batava* |
| h35 | 6 | *S. abbreviata* |
| h36 | 25 | *S. contigua* |
| h37 | 1 | *S. contigua* |
| h38 | 2 | *S. contigua* |
| h39 | 1 | *S. sulphuripes* |
| h40 | 2 | *S. sulphuripes* |
| h41 | 1 | *S. sulphuripes* |
| h42 | 1 | *S. pyrrhina* |
| h43 | 3 | *S. shirchan* |
| h44 | 33 | *S. novaeangliae* |
| h45 | 1 | *S. novaeangliae* |
| h46 | 1 | *S. novaeangliae* |
| h47 | 1 | *S. novaeangliae* |
| h48 | 1 | *S. novaeangliae* |
| h49 | 1 | *S. novaeangliae* |
| h50 | 1 | *S. novaeangliae* |
| h51 | 1 | *S. novaeangliae* |

| **Supp. Table S6.** The average population membership probabilities of the analyzed *Sphaerophoria scripta* geographic samples to the most probable number of genetic clusters retrieved from BAPS admixture analysis using *COI* mtDNA and allozyme loci. NLD - the Netherlands (the Hague), DEU - Germany (Berlin), SVN - Slovenia (Bled), BIH - Bosnia and Herzegovina (Banja Luka), GRC - Greece (Araxos) | | | | | |
| --- | --- | --- | --- | --- | --- |
|  | **K1** | **K2** | **K3** | **K4** | **K5** |
| ***COI* mtDNA** |  |  |  |  |  |
| NLD | 0.836 | 0.079 | 0.085 |  |  |
| DEU | 0.805 | 0.102 | 0.093 |  |  |
| SVN | 0.846 | 0.075 | 0.078 |  |  |
| BIH | 0.499 | 0.429 | 0.071 |  |  |
| GRC | 0.483 | 0.320 | 0.197 |  |  |
| **Allozyme loci** |  |  |  |  |  |
| NLD | 0.056 | 0.056 | 0.444 | 0.000 | 0.444 |
| DEU | 0.200 | 0.000 | 0.400 | 0.200 | 0.200 |
| SVN | 0.000 | 0.190 | 0.095 | 0.381 | 0.333 |
| BIH | 0.389 | 0.222 | 0.167 | 0.000 | 0.222 |
| GRC | 0.136 | 0.409 | 0.091 | 0.136 | 0.227 |

| **Supp. Table S7.** The mean cluster membership coefficients of the analyzed *Sphaerophoria scripta* geographic samples retrieved from STRUCTURE analysis using allozyme loci. Methods by Evanno et al. (2005) and Pritchard et al. (2000) retrieved K=4 and K=5 as the most probable values of *K*, respectively, therefore coefficients are summarized for both scenarios. The values are averaged over the ten runs for their respective optimal K. NLD - the Netherlands (the Hague), DEU - Germany (Berlin), SVN - Slovenia (Bled), BIH - Bosnia and Herzegovina (Banja Luka), GRC - Greece (Araxos) | | | | | |
| --- | --- | --- | --- | --- | --- |
| **Best K by Evanno et al. (2005)** | **K1** | **K2** | **K3** | **K4** |  |
| NLD | 0.442 | 0.094 | 0.419 | 0.045 |  |
| DEU | 0.256 | 0.052 | 0.392 | 0.300 |  |
| SVN | 0.265 | 0.207 | 0.103 | 0.425 |  |
| BIH | 0.459 | 0.138 | 0.181 | 0.223 |  |
| GRC | 0.295 | 0.412 | 0.101 | 0.192 |  |
| **Best K by Pritchard et al. (2000)** | **K1** | **K2** | **K3** | **K4** | **K5** |
| NLD | 0.167 | 0.076 | 0.400 | 0.027 | 0.331 |
| DEU | 0.224 | 0.035 | 0.343 | 0.227 | 0.171 |
| SVN | 0.032 | 0.191 | 0.109 | 0.391 | 0.277 |
| BIH | 0.458 | 0.109 | 0.161 | 0.072 | 0.199 |
| GRC | 0.149 | 0.386 | 0.096 | 0.161 | 0.208 |

| **Supp. Table S8.** The results of the analyses of molecular variance (AMOVA) for the analysed geographic samples of *Sphaerophoria scripta*. | | | | |
| --- | --- | --- | --- | --- |
|  | Variation sources | Variance components | Variation (%) | Fixation indices |
| ***COI* mtDNA** | Among geographic samples | -0.021 | -1.58 |  |
|  | Within geographic samples | 1.366 | 101.58 | Φ_ST_=-0.02  P=0.74 |
| **Allozymes** | Among geographic samples | 0.043 | 8.05 |  |
|  | Within geographic samples | 0.492 | 91.95 | F_ST_=0.08  P=0.02 |

| **Supp. Table S9.** Pairwise Fst values between the pairs of the geographic samples of *Sphaerophoria scripta* analysed using allozyme loci. Statistically significant comparisons (P<0.05) are bolded. NLD - the Netherlands (the Hague), DEU - Germany (Berlin), SVN - Slovenia (Bled), BIH - Bosnia and Herzegovina (Banja Luka), GRC - Greece (Araxos) | | | | | |
| --- | --- | --- | --- | --- | --- |
|  | NLD | DEU | SVN | BIH | GRC |
| NLD |  |  |  |  |  |
| DEU | 0.05 |  |  |  |  |
| SVN | 0.09 | 0.02 |  |  |  |
| BIH | -0.03 | 0.06 | **0.23** |  |  |
| GRC | 0.03 | 0.06 | **0.13** | 0.09 |  |


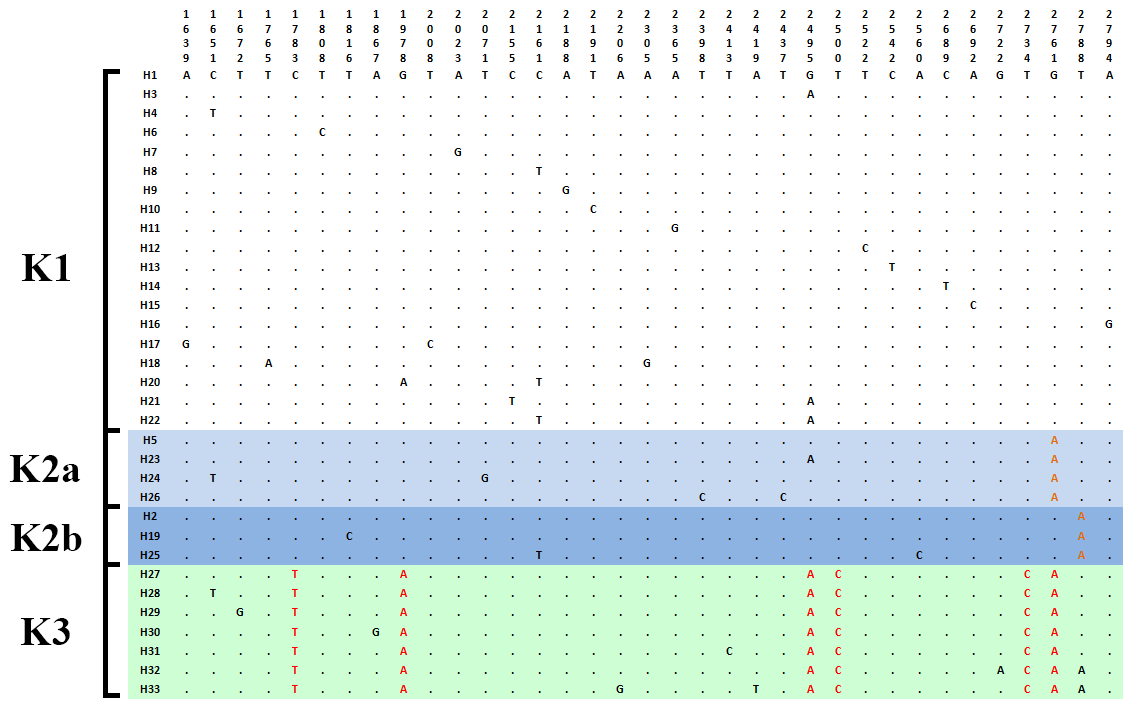


**Supp. Fig. S1.** The variable positions (relative to the reference mitochondrial genome of *Episyrphus balteatus*; GenBank accession code: KU351241) among *Sphaerophoria scripta* *COI* mtDNA haplotypes (1209 bp-long fragment) and the respective clusters recognized by BAPS analysis. Diagnostic positions defining members of clusters K2 and K3 are distinctly colored
